# SUMO promotes longevity and maintains mitochondrial homeostasis during ageing in *Caenorhabditis elegans*

**DOI:** 10.1038/s41598-020-72637-9

**Published:** 2020-09-23

**Authors:** Andrea Princz, Federico Pelisch, Nektarios Tavernarakis

**Affiliations:** 1 Department of Biology, University of Crete, Heraklion, Greece; 2 Institute of Molecular Biology and Biotechnology, Foundation for Research and Technology-Hellas, Heraklion, Greece; 3 Centre for Gene Regulation and Expression, Sir James Black Centre, School of Life Sciences, University of Dundee, Dundee, Scotland, UK; 4 Department of Basic Sciences, Faculty of Medicine, University of Crete, Heraklion, Greece

**Keywords:** Cell biology, Organelles, Post-translational modifications, Ageing

## Abstract

The insulin/IGF signalling pathway impacts lifespan across distant taxa, by controlling the activity of nodal transcription factors. In the nematode *Caenorhabditis elegans*, the transcription regulators DAF-16/FOXO and SKN-1/Nrf function to promote longevity under conditions of low insulin/IGF signalling and stress. The activity and subcellular localization of both DAF-16 and SKN-1 is further modulated by specific posttranslational modifications, such as phosphorylation and ubiquitination. Here, we show that ageing elicits a marked increase of SUMO levels in *C. elegans*. In turn, SUMO fine-tunes DAF-16 and SKN-1 activity in specific *C. elegans* somatic tissues, to enhance stress resistance. SUMOylation of DAF-16 modulates mitochondrial homeostasis by interfering with mitochondrial dynamics and mitophagy. Our findings reveal that SUMO is an important determinant of lifespan, and provide novel insight, relevant to the complexity of the signalling mechanisms that influence gene expression to govern organismal survival in metazoans.

## Introduction

Organismal homeostasis is relentlessly challenged by various external and internal stressors. Over the course of life, homeostasis gradually declines and finally collapses, leading to death. Studies in organisms ranging from microbes to humans have culminated in the identification of multiple hallmarks of the ageing process and numerous regulatory factors that influence lifespan^1,2^. Amongst these, the insulin/IGF-1 pathway has a major role in lifespan determination^3,4^; and is evolutionarily conserved from worms to mammals^5^. In the nematode *Caenorhabditis elegans*, it entails the activation of insulin receptor DAF-2 (dauer formation abnormal), by insulin-like peptides. The activated receptor triggers a series of phosphorylation events in the cytoplasm, including the phosphorylation of the FOXO transcription factor, DAF-16. The phosphorylated form of DAF-16 is retained in the cytoplasm. Under stress conditions (heat shock, starvation, oxidative stress) DAF-16 is no longer phosphorylated and translocates to the nucleus, where it activates the transcription of genes responsible for development, longevity, re-wiring of metabolism, and stress responses^6,7^. Importantly, other stress responsive transcription factors such as SKN-1, function together with DAF-16. SKN-1 (skinhead) is the orthologue of the mammalian NRF2 (Nuclear factor erythroid 2-related factor 2) and has a main role in oxidative stress response^8^.

The functional decline of mitochondria during ageing is another major determinant of lifespan. The ageing process brings about the accumulation of dysfunctional mitochondria, which exhibit increased ROS production, accrual of mitochondrial DNA, and less efficient ATP production^1^. In addition, mitochondrial dynamics is also altered over the course of lifespan^9^. The proper balance of mitochondrial biogenesis and mitochondrial clearance by organelle-specific autophagy (mitophagy) is a prerequisite of healthy ageing. DAF-16 and SKN-1 are key regulators of these two processes^10^. The activity of these transcription factors is modulated by many targeted posttranslational protein modifications, including phosphorylation, methylation, and ubiquitination^11–13^. We sought to investigate whether other modes of posttranslational modification influence the activity of these nodal transcription factors, under normal and stress conditions.

SUMOylation, the attachment of a small ubiquitin like modifier (SUMO) to a target protein, is a posttranslational modification that plays pivotal roles in fundamental cellular processes including DNA damage responses, mitochondrial dynamics, development and senescence, among others^14,15^. In close analogy to ubiquitination, regulatory SUMOylation requires a heterodimeric E1 activating enzyme, an E2 conjugation enzyme, an E3 SUMO ligase and a SUMO protease. The *C. elegans* genome encodes a sole SUMO gene, *smo-1*, which makes it an expedient model organism to dissect its diverse functions. SUMOylation has been mainly studied in the context of development in *C. elegans*, and has been implicated in embryonic, vulval and muscle development, and in DNA damage responses^16–27^. Recently, SUMOylation of a specific RNA binding protein (CAR-1) in the germline has been linked to organismal ageing^28^. Notably, the level of SUMO1 modified proteins has been shown to progressively increase during ageing in blood plasma and cortical lysates of mice^29^. However, a causative relationship between SUMOylation and ageing has not been established.

Here, we uncover a direct link between SUMO and the regulation of ageing, in *C. elegans*. Perturbation of SUMO levels causes alterations in lifespan and mitochondrial homeostasis in a DAF-16/FOXO and SKN-1/NRF2 dependent manner. Reduced *smo-1* expression leads to shortened lifespan, coupled with impaired mitochondrial ATP and ROS production, mitophagy defects and over-activation of stress response genes. In addition, we demonstrate for the first time that DAF-16 is SUMOylated, and that this modification represses the transcriptional activity of DAF-16.

## Materials and methods

### *C. elegans* strains and genetics

We followed standard procedures for maintenance of *C. elegans* strains and transgenic lines^30^. Animals were grown at 20 °C unless noted otherwise. The following strains were used for this study: N2: wild type Bristol isolate, CB1370: *daf-2(e1370)III*, MQ887: *isp-1(qm150)IV*, CF1038: *daf-16(mu86)I*, KX15: *ife-2(ok306)X*, SJ4143: N2;*Is*[p_*ges-1*_mtGFP], CL2166: N2;*Is*[p_*gst-4*_GFP], CF1553: N2;*Is*[p_*sod-3*_GFP], TJ356: N2;Is[p_*daf-16*_DAF-16a/b::GFP;*rol-6(su1006)*], FGP14: *unc-119(ed3)*; *fgpIs35*[*unc-119*(+);p_*smo-1*_*::6xHis::smo-1::smo-1 3*′* UTR*], VP303: *rde-1(ne219)V;kbIs7*[p_*nhx-2*_*::rde-1;rol-6(su1006)*], NR350: *rde-1(ne219)V*; *kzIs20*[p_*hlh-1*_*::rde-1*;p_*sur-5*_::NLS::GFP], NR222: *rde-1(ne219)V;kzIs9*[p_*lin-26*_::NLS::GFP;p_lin-26_*::rde-1;rol-6(su1006)*], TU3401: *sid-1(pk3321)V;uIs69*[p_*myo-2*_::mCherry;p_*unc-119*_*::sid-1*], NL2099: *rrf-3(pk1426)II*, JRIS1: N2; Is[p_*rpl-17*_HyPer]..

### Molecular cloning

To create a p_*smo-1*_DsRed::SMO-1 construct, we utilized the pPD95.77(DsRed) construct, previously generated in the lab. Briefly, GFP was excised from pPD95.77 (GFP) vector using the AgeI/EcoRI restriction enzymes, and was replaced with the fragment coding DsRed, isolated from p_*col-12*_DsRed^31^. We amplified the *smo-1* ORF from *C. elegans* genomic DNA, using the following primer pair: 5′-GAATTCCAACATGGCCGATGATGCAG-3′ and 5′-GGATCCCGTTTATAGCGGGAGTCTC-3′ and the 560 bp product was first subcloned into pCRII-TOPO vector (Invitrogen). Using the restriction enzyme EcoRI, the fragment was excised and inserted into pPD95.77 (DsRed) and was sequenced to check for possible mutations and orientation. The correct construct was introduced to *unc-119(ed3)* worms, together with pRH21^32^, using biolistic transformation. To generate the p_*vha-6*_SMO-1 construct, we amplified the *smo-1* ORF from *C. elegans* genomic DNA, using the following primer pair: 5′-GTTTCAGAGACTCCCGCTATAAAC-3′ and 5′-GCTAGCCGAAGAGTTTATTTGTAAG-3′ and the 570 bp product was subcloned into pCRII-TOPO vector. Using the restriction enzymes BamHI/NheI, the fragment was inserted in pPD96.52 vector, containing the *myo-3* promoter. The promoter was removed, using the HindIII/XbaI restriction enzymes and the 950 bp coding sequence of *vha-6* promoter was inserted from pCRII-TOPO. To amplify the *vha-6* promoter from *C. elegans* genomic DNA, we used the following primer pair: 5′-GAGAAGATGTGATGAGATGGAGAGAAAG-3′ and 5′-TGAGCACTTTACAGTTCTTGTTGATTTG-3′. The construct, together with the pRH21 plasmid, was introduced to *unc-119(ed3)* worms, using biolistic transformation. To generate the p_*rab-3*_SMO-1 construct, we amplified the *smo-1* ORF from *C. elegans* genomic DNA, using the following primer pair: 5′-CCCGGGGTTTCAGAGACTC-3′ and 5′-GGTACCCGAAGAGTTTATTTGTAAG-3′ and the 580 bp product was subcloned into pCRII-TOPO vector. We inserted the Xma/KpnI fragment in pPD95.77, containing the *rab-3* promoter. The construct and the pRH21 plasmid were introduced to *unc-119(ed3)* worms, using biolistic transformation. For the generation of *smo-1*, *ubc-9* , *ulp-1*, *ulp-2*, *ulp-4* and *ulp-5* RNAi construct, the following primer pairs were used: 5′-TAAACGATGGCCGATGATGC-3′ and 5′-GGATCCCGAAAGAGTTTATTTGTAAG-3′, 5′-ATGTCGGGAATTGCTGCAGG-3′ and 5′-GTCCAGAAGCAAATGCTCGAGTAG-3′, 5′-CCACAGTTGTTACAGAACAAG-3′ and 5′-GGATCCCACATTAGTTTCTCTGCTTC-3′, 5′-ATGAGTGATTCAACTCAAATGGAGG-3′ and 5′-CATACAGCAGTTGCCACAAACG-3′, 5′-ATGGAAGTGTCAACGTCTTACTGTACG-3′ and 5′-GATTTTTGCTGTCTCCAGGAGAAG-3′, 5′-ATGCCTCATCCTAAGCTCACTCC-3′ and 5′-GCCTGGTTCATTCAAAAAAATACAG-3′, respectively. The resulted fragments were cloned into the vector pL4440 and the constructs were transformed into HT115 (DE3) *Escherichia coli* strain, which is defective for RNase III. Bacteria carrying the pL4440 empty vector alone were used for control experiments. RNAi constructs against *daf-2*, *eat-3*, *daf-16*, *skn-1, drp-1, fzo-1* have been described previously^10,33^. Double RNAi against *skn-1* and *smo-1* was constructed by digesting the *skn-1(RNAi)* and *smo-1(RNAi)* plasmids with BamHI restriction enzyme and ligating the *smo-1* fragment (~ 1000 bp) into *skn-1(RNAi)* plasmid, treated with Alkaline Phosphatase, Calf Intestinal (CIP, New England Biolabs).

### Epifluorescence and confocal microscopy

Transgenic worms were placed in a drop of 10 mM levamisole on a microscope slide and sealed with a cover slip. Images were taken with a Zeiss AxioImager Z2 epifluorescence microscope. At least 25 worms were imaged and quantified per condition; each experiment was repeated at least three times. Mean fluorescence intensity was measured using the software Image J. The nuclear/cytoplasmic ratio of p_*daf-16*_DAF-16::GFP was measured as follows: we measured the mean fluorescence intensity of a nucleus and the intensity of the same selection area of the corresponding cytoplasm. These two numbers were divided to get the ratio. For confocal microscopy, animals were immobilized on a 5% agarose pad, in a 5 μl drop of Nanobeads (Nanobeads NIST Traceable Particle Size Standard 100 nm, Polysciences). Images were taken with an LSM710 Zeiss confocal microscope, Axio-observer Z1.

### Western blot analysis

For the analysis of SUMO conjugated proteins during ageing, synchronized animals were collected in M9 buffer and after a short spin, the buffer was exchanged to the lysis buffer (50 mM Tris–HCl pH: 7.4, 150 mM NaCl, 1 mM EDTA, 1% Triton X-100, 1 mM PMSF, 30 mM NEM) supplemented with protease inhibitor cocktail (cOmplete mini protease inhibitors cocktail tablets—ROCHE) just before use. Animals were frozen at − 80 °C and then boiled at 95 °C together with 1X Laemmli sample buffer (70 mM SDS, 1.5 mM bromophenol blue, 0.8% glycerol, 10 mM Tris–HCl pH: 6.8, 100 mM DTT). The lysates were spun down at 4 °C, then loaded and separated by 4–12% Bis–Tris gel and transferred to PVDF membrane (Amersham-GE Healthcare). The membrane was blocked using 5% non-fat milk for 1 h and then incubated overnight with anti-SUMO antibody^34^ (Developmental Studies Hybridoma Bank (DSHB) Cat# SUMO 6F2, RRID:AB_2618393, 1:250) and with anti-α-tubulin antibody (DSHB Cat# AA4.3, RRID:AB_579793, 1:5000) in 5% non-fat milk at 4 °C. The next day the membrane was washed with 1XPBS-T (0.1% Tween 20) and incubated with horseradish peroxidase-conjugated secondary antibody for 1 h at room temperature. After washes with 1XPBS-T, the membrane was developed by chemiluminescence (Supersignal chemiluminescent substrate pico and femto, Thermo Fisher Scientific).

For the analysis of SUMO conjugated proteins upon *smo-1(RNAi)* and SMO-1 overexpression (FGP14 strain), animals were washed and collected from two plates in M9 buffer with 0.1% Triton-X 100. Animals were washed one time with M9 buffer with 0.1% Triton-X 100 and transferred to a new tube. 200 μl of 40% trichloroacetic acid (TCA) (Sigma-Aldrich) and 300 μl glass beads (Sigma-Aldrich) were added to each sample. Samples were lysed by using a Beadbeater (Biospec), for 3 min (1 min on–1 min off) in a cold room. Lysates were transferred to a new tube and the beads were washed with 5% TCA and mixed with the lysates. Samples then were centrifuged for 15 min at 13,000 rpm at 4 °C. The pellet was washed three times with 500 μl chilled acetone. Pellets were dried at room temperature and resuspended in 100 μl 1X Sample Reducing Agent (Thermo Fisher Scientific). Following a 5 min incubation at 70 °C, samples were sonicated 2× for 10 s (12% amplitude). Samples were incubated again at 70 °C and then centrifuged for 10 min at 11,000 rpm. Samples were transferred to a new tube and loaded onto a 4–12% Bis–Tris gel for separation, transferred to a nitrocellulose membrane (Amersham-GE Healthcare). The membrane was blocked in a blocking solution (Invitrogen) for 1 h and then incubated overnight with anti-SUMO antibody (sheep)^35^ (1:1000) and with anti-α-tubulin antibody (DSHB Cat# AA4.3, RRID:AB_579793, 1:5000) in 3% BSA at 4 °C. The next day the membrane was washed with 1XPBS-T (0.1% Tween 20) and incubated with AlexaFluor 647 secondary antibody for 1 h at room temperature. After washes with 1XPBS-T, the membrane was analyzed with Amersham Typhoon Biomolecular Imager (GE Healthcare).

### Mitochondria isolation

Age matched animals were collected in M9 buffer, and incubated at 4 °C with rotation in the presence of 10 mM DTT. To remove the DTT from the sample, three washes were performed with M9. Worms were homogenized in incubation buffer [50 mM Tris–HCl pH:7.4, 210 mM mannitol, 70 mM sucrose, 0.1 mM EDTA, 2 mM PMSF, cOmplete mini protease inhibitors cocktail (ROCHE)] with 100 strokes in a 3 ml Potter–Elvehjem homogenizer with PTFE pestle and glass tube (Sigma-Aldrich). The lysate was centrifuged at 200*g* for 1 min. The pellet was subjected to another round of homogenization. The lysate was combined with the supernatant from the previous centrifugation step, and they were centrifuged together for 1 min at 200*g*. The supernatant was centrifuged again at 12,000*g* for 5 min. We kept the supernatant as the cytoplasmic fraction and resuspended the pellet/mitochondrial fraction in incubation buffer. Samples were analysed on 4–12% SDS-PAGE gradient gels with anti-SUMO (DSHB Cat# SUMO 6F2, RRID:AB_2618393), anti-MTCO1 (Abcam [1D6E1A8] (ab14705)) and anti-α-tubulin (DSHB Cat# AA4.3, RRID:AB_579793).

### DAF-16 and SKN-1 purification

We codon optimized the sequence of isoform “a” of DAF-16 (UniProt number: O16850) and isoform “c” of SKN-1 (UniProt number: P34707) and inserted into a vector suitable for bacterial expression, pHis-TEV-30a^36^. This vector also contains an N terminal 6xHis-MBP tag and a TEV cleavage site. The construct was transformed to BL21 Rosetta *E. coli* strain for protein expression. Bacterial cultures were grown at 37 °C until OD_600_ = 0.8, then cooled down and induced with 1 mM IPTG at 20 °C for 4 h for DAF-16 expression and with 0.1 mM IPTG at 37 °C for 4 h for SKN-1 expression. The cells were pelleted by centrifugation (4500 rpm, 30 min, 4 °C) and resuspended in 50 ml lysis buffer (50 mM Tris–HCl, 0.5 M NaCl, 10 mM imidazole, pH 7.5) supplemented with 0.5 mM TCEP and cOmplete mini protease inhibitors cocktail tablets – ROCHE. Cells were lysed by sonication (Digital Sonifier, Branson) for 5X 20 s pulses at 50% amplitude with 20 s cooling period between pulses. Cell lysate was centrifuged (15,000 rpm, 45 min, 4 °C) to clear the sample from any insoluble material and supernatant was loaded onto a 2 ml Ni–NTA column (Qiagen) pre-equilibrated with lysis buffer. After sample binding, the column was washed with ten column volume of lysis buffer and with ten column volume of lysis buffer containing 30 mM imidazole. The protein was eluted from the column with elution buffer (50 mM Tris–HCl, 150 mM NaCl, 150 mM imidazole, 0.5 mM TCEP). DAF-16 was further purified by size exclusion chromatography with Superose 6 Increase 10/300 column (GE Healthcare). Purified and concentrated protein was aliquoted and stored at − 80 °C.

### In vitro SUMOylation assays

Conjugation assays contained 50 mM Tris–HCl, 0.5 mM TCEP, 5 mM MgCl_2_, 2 mM ATP, 5 μg SUMO, 0.5 μg SUMO-Alexa Fluor 680, 60 ng SAE1/SAE2 (SUMO E1), 5 or 20 ng UBC-9, 10, 50 or 100 ng GEI-17 and 5 μg DAF-16, SKN-1 or MBP. Reactions were incubated at 30 °C for 4 h and they were run parallel on 4–12% Bis–Tris (better visualization of free SUMO) and 3–8% Tris–Acetate gels (better visualization of SUMO modified proteins). Gels were analyzed by Coomassie staining and Amersham Typhoon Biomolecular Imager (GE Healthcare)^35^.

### Mitochondrial imaging

For TMRE (tetramethylrhodamine, ethyl ester) (Sigma) staining, age-matched animals were placed overnight on an RNAi plate containing 150 nM TMRE and the next morning placed in a 10 mM levamisole drop on a microscope slide, sealed with a cover slip. Images were taken with a Zeiss AxioImager Z2 epifluorescence microscope. For mitochondrial ROS staining, synchronized animals were placed on RNAi plates containing 150 nM MitoTracker Red CMXROS (Thermo Fisher Scientific) overnight and the next morning were mounted on microscope slides in a 10 mM levamisole drop, sealed with a cover slip to assess mitochondrial ROS production. Images were taken with a Zeiss AxioImager Z2 epifluorescence microscope. Images were quantified using the software Image J. For paraquat treated TMRE staining, age-matched animals were placed on RNAi plates containing 4 mM paraquat for 1 day, and the next day were transferred to a fresh RNAi plate containing 4 mM paraquat and 150 nM TMRE. The next morning the animals were placed on microscope slides in a 10 mM levamisole drop, sealed with a cover slip. Images were taken with a Zeiss AxioImager Z2 epifluorescence microscope. Images were quantified using the software Image J. For monitoring mitophagy we used the previously described mitochondrial targeted Rosella biosensor in body wall muscle cells of the animals^10^, with a minor modification. We created a new transgenic line with biolistic transformation and fed these animals with the test RNAi constructs. Animals were mounted on 5% agarose pads in a 10 mM levamisole drop and sealed with a cover slip. Images were taken with an Invitrogen EVOS FL Auto 2 Cell Imaging System. Images were quantified using the software Image J.

### Messenger RNA quantification

Total RNA was extracted from worms, using Trizol (Sigma). cDNA was synthesized using the iScript kit (BioRad). Quantitative Real Time PCR was performed using a Bio-Rad CFX96 Real-Time PCR system, and was repeated three times. The following primer pairs were used to quantify the expression of genes: for *ges-1*: 5′-TCGCCAAGAGGTATGCTTCACAAG-3′ and 5′-TGCTGCTCCTGCACTGTATCCC-3′, for *gst-4*: 5′-GGCAAGAAAATTTGGACTC-3′ and 5′-GCCAAGAAATCATCACGGGC-3′, for *sod-3*: 5′-ATTGCTCTCCAACCAGCGC-3′ and 5′-GGAACCGAAGTCGCGCTTAA-3′, for *smo-1*: 5′-AAGATCAAGGTCGTTGGACAGGAC-3′ and 5′-CTAGAATCCGCCCAGCTGCT-3′.

### ATP measurements

To quantify intracellular ATP levels, we followed the protocol described here^37^. In short, 100 age matched animals were collected in 50 µl of M9 buffer and frozen at − 80 °C. Frozen worms were boiled at 95 °C for 15 min. After a 10 min centrifugation step at 14,000 rpm at 4 °C, the supernatant was transferred to a fresh tube and diluted tenfold before measurement. ATP content was determined by using the Roche ATP bioluminescent assay kit HSII (Roche Applied Science) and a TD-20/20 luminometer (TurnerDesigns). ATP levels were normalized to total protein content.

### Oxygen consumption rate measurement

To determine oxygen consumption rates, we followed a previously described protocol^38^. In short, 4-day-old adult animals were collected in 1 ml M9, and transferred to the chamber of Oxygraph (Hansatech Instruments). Measurements were performed for 15 min at 20 °C. The oxygen consumption rate was obtained by the slope of the straight portion of the plot. The animals were recovered after measurement and were subjected to sonication and protein determination. The oxygen consumption rates were normalized to total protein content.

### Mitochondrial DNA quantification

mtDNA was quantified using quantitative real time PCR as described previously^39^. 50 worms were collected per condition, lysed and diluted tenfold before performing the PCR reaction. The following primer set was used for mtDNA (*mito-1*): 5′-GTTTATGCTGCTGTAGCGTG-3′ and 5′-CTGTTAAAGCAAGTGGACGAG-3′. The results were normalized to genomic DNA using the following primers specific for *ama-1*: 5′-TGGAACTCTGGAGTCACACC-3′ and 5′-CATCCTCCTTCATTGAACGG-3′. Quantitative PCR was performed using a Bio-Rad CFX96 Real-Time PCR system, and was repeated three times.

### Lifespan analysis

Experiments were carried out at 20 °C, unless noted otherwise. Animals were synchronized by placing 7–8 gravid adults on control RNAi plates for overnight egg laying and they were removed the next morning. L4 larvae were placed on experimental plates (containing 2 mM IPTG and seeded with HT115 (DE3) bacteria comprising the control vector (pL4440) or the test RNAi construct) and they were transferred to a fresh plate every 2–3 days. Animals were scored for survival with movement provoking touch every second day. Those who crawled out of the plate or died due to internal egg-hatching were considered censored and incorporated into the dataset as such. Each lifespan assay was repeated at least two times and figures represent typical assays. Lifespan assays on NAC (*N*-acetyl cysteine) plates (10 mM final concentration) were performed only one time. Statistical analysis was performed using the Prism software package (version 7; GraphPad Software; https://www.graphpad.com), and the product-limit method of Kaplan and Meier.

### Survival assays

For oxidative stress assays we grew synchronized animals until day 6 of adulthood on control or *smo-1(RNAi)* plates. At day 6 animals were transferred to control or *smo-1(RNAi)* plates containing paraquat (methyl viologen dichloride hydrate, Sigma) in 2 mM final concentration. Additionally, bacteria were killed with UV before adding paraquat on the plates in order to prevent interference arising from bacterial metabolism.

For acute heat stress survival the animals were grown until day 4 of adulthood on control or *smo-1(RNAi)* plates at 20 °C. At day 4 of adulthood, animals were subjected to 37 °C for 2 h and then placed back to 20 °C. In both stress conditions animals were scored for survival with movement provoking touch every day. Those who crawled out of the plate or died due to internal egg-hatching were considered censored and incorporated into the dataset as such. Each stress survival assay was repeated at least two times and figures represent typical assays. Statistical analysis was performed using the Prism software package (version 7; GraphPad Software; https://www.graphpad.com), and the product-limit method of Kaplan and Meier.

### Statistical analysis

Statistical analyses were carried out using the Prism software package (version 7; GraphPad Software; https://www.graphpad.com) and the Microsoft Office 2010 Excel software package (Microsoft Corporation, Redmond, WA, USA). Mean values were compared using unpaired t-tests or one-way ANOVA.

## Results

### SUMOylation modulates ageing in *C. elegans*

**Figure 1:**
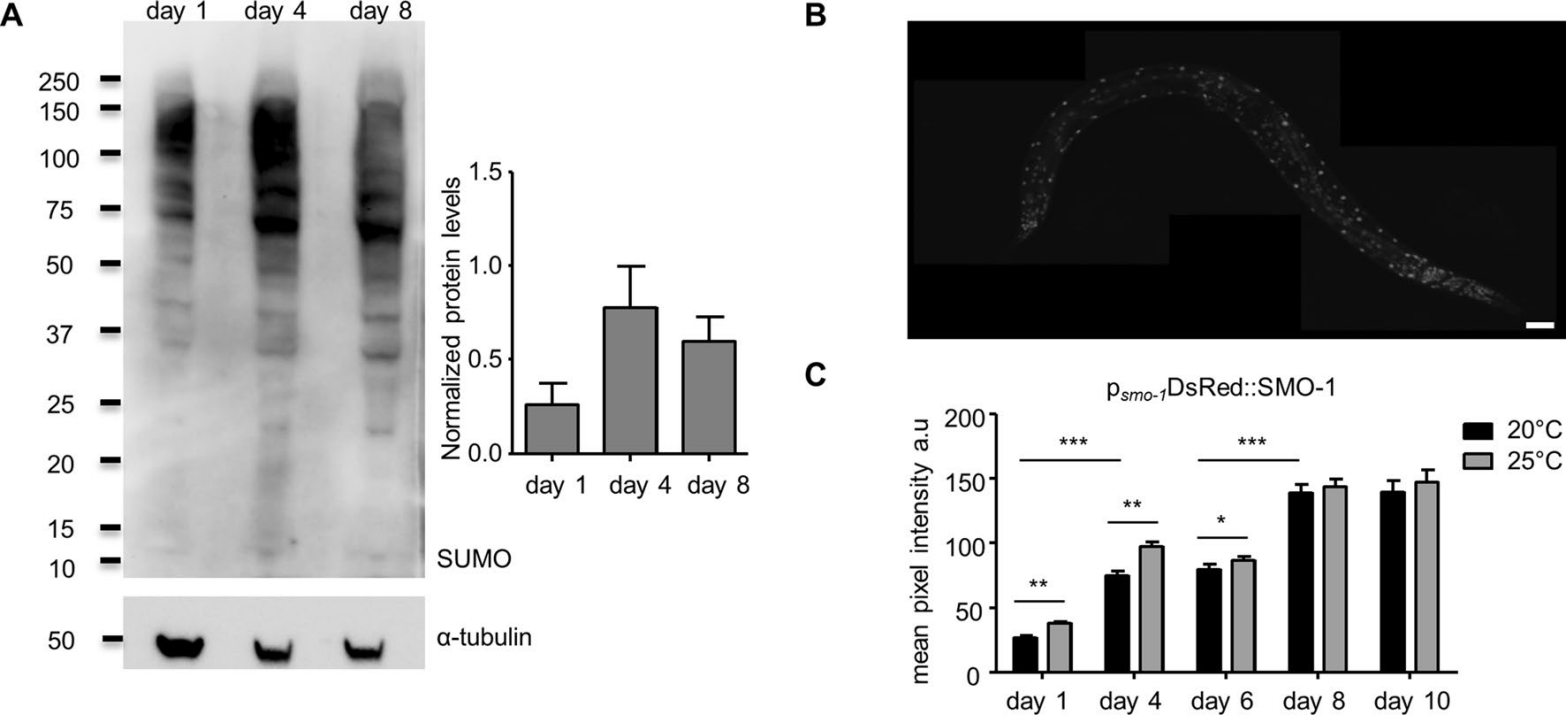
SUMO levels are increasing during ageing. (**A**) Western blot analysis of SUMO protein levels in day 1, 4 and 8 wild type lysates (n = 500 worms/sample, N = 4, day 1 vs day 4: p = 0.103, day 1 vs day 8: p = 0.119). Protein levels were normalized to α-tubulin. Error bars, S.E.M. (**B**) p_*smo-1*_DsRed::SMO-1 expression pattern, scale bar: 40 μm. The image was acquired using a × 20 objective lens. (**C**) The expression of p_*smo-1*_DsRed::SMO-1 is increasing during ageing and when the animals are grown at 25 °C. (n = 50, *p < 0.05, **p < 0.01, ***p < 0.001, unpaired t-test). Error bars, S.E.M.

To examine SUMOylation over the course of ageing in *C. elegans*, we performed Western blot analysis, using age matched, wild type animals. We find that the amount of SUMO conjugated proteins peaks at day 4 of adulthood in *C. elegans* (Fig. 1A). To assess SUMO expression, we generated transgenic animals, expressing a full-length, DsRed-tagged SMO-1 reporter fusion (p_*smo-1*_DsRed::SMO-1; Fig. 1B). We observed nuclear localization of SUMO in all tissues (e.g. neurons, seam cells, intestine and muscles; Fig. 1B). SMO-1 expression increases during ageing and upon a temperature shift to 25 °C (Fig. 1C). Thus, SMO-1 expression is progressively increasing with age and under mild thermal stress, in *C. elegans*.

**Figure 2:**
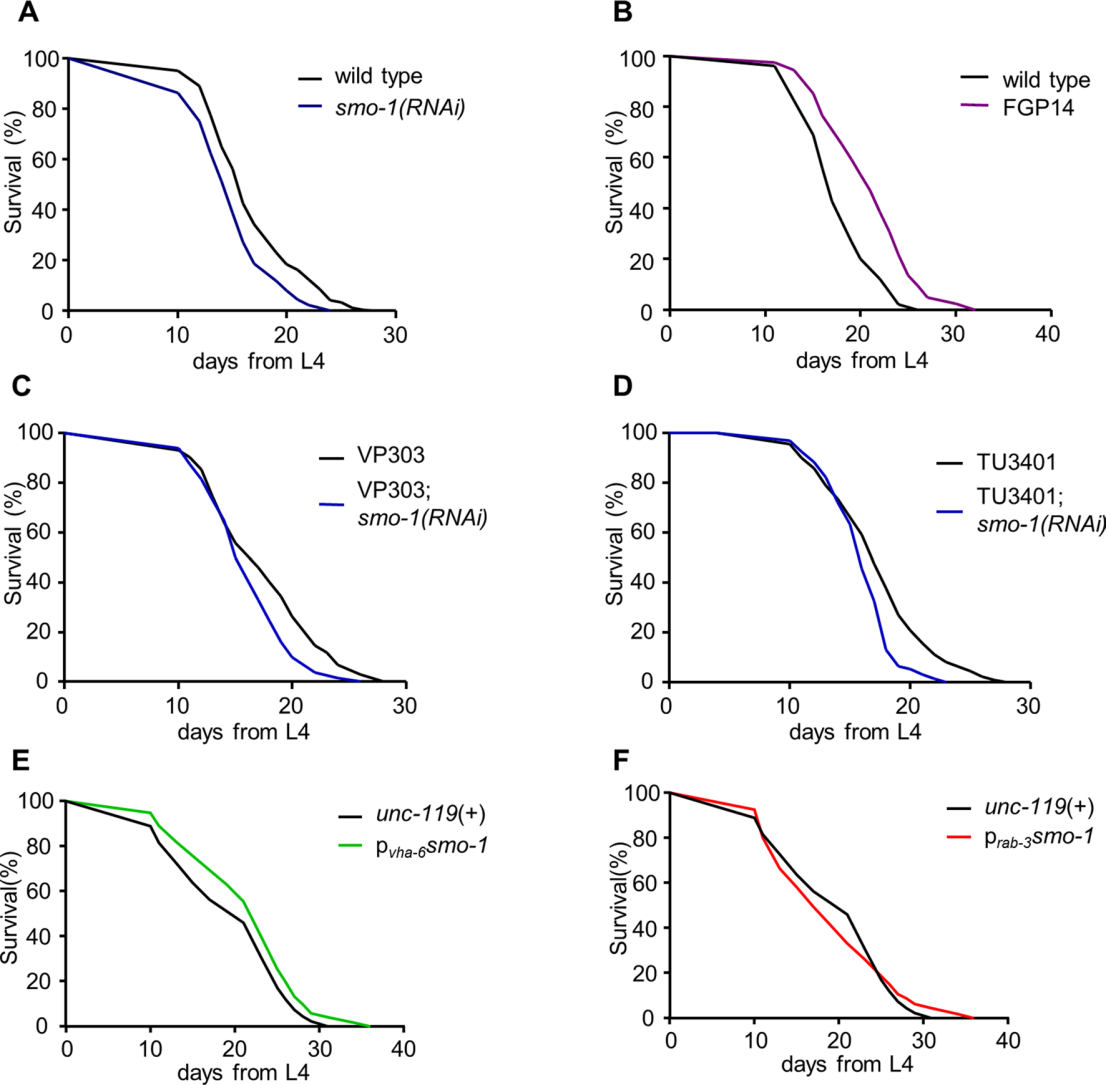
SUMO modulates lifespan through the intestine and nervous system. (**A**, **B**) Knockdown of *smo-1* shortens the lifespan of wild type animals, while overexpression of *smo-1* extends lifespan. (**C**) Intestine specific *smo-1(RNAi)* shortens lifespan. (**D**) Neuron specific *smo-1(RNAi)* reduces lifespan. (**E**) Intestine specific *smo-1* overexpression extends lifespan. (**F**) Neuron specific *smo-1* overexpression does not have a significant effect on lifespan. Lifespan assays were carried out at 20 °C. Lifespan values are given in Table S1.

SUMOylation is essential during development^40^. Consequently, mutation of *smo-1* causes embryonic lethality^41,42^. To examine the physiological significance of SUMO accumulation during ageing, we reduced *smo-1* expression post-developmentally, at the end of the L4 larval stage by RNAi (Fig. S1A). We observed significant reduction of animal lifespan (Fig. 2A). By contrast, downregulation of the 4 SUMO proteases (ubiquitin-like proteases), encoded in the *C. elegans* genome (*ulp-1-2, ulp-4-5*), did not alter the lifespan of wild type animals (Fig. S1B,C), under normal conditions. However, reduced expression of *ulp-1* resulted in a prolonged lifespan at 25 °C (Fig. S1D). Importantly, *smo-1* overexpression extended lifespan (FGP14 strain, Pelisch and Hay, 2016; p_*smo-1*_*::6xHis::smo-1*::*smo-1* 3′ UTR; Figs. S1A, 2B). These findings indicate that SUMO is a modulator of ageing in *C. elegans*.

Previous studies have established the prominent contribution of specific tissues in the control of the ageing process^43^. To examine whether SUMO promotes longevity in a tissue specific manner, we targeted *smo-1* expression in different tissues by RNAi. We utilized transgenic strains carrying a mutation in *rde-1* gene (RNAi defective), which encodes an Argonaute protein family member that is required for RNA interference^44^. A wild type version of *rde-1* is then re-introduced to the animals in a tissue specific manner, restoring RNAi capacity in that tissue. Using this approach, we assessed the effects of SUMO downregulation in the intestine (VP303 strain), hypodermis (NR222 strain) and muscles (NR350 strain). Only intestine specific *smo-1(RNAi)* resulted in shorter lifespan, while knockdown of *smo-1* in hypodermal and muscle tissues had no effect (Fig. 2C, Fig.S1E,F). We also investigated the consequences of neuron-specific SUMO deficiency. The nervous system of *C. elegans* is mostly resistant to RNAi, but specific neurons (amphids and phasmids) are susceptible^45^. To probe neuron specific SUMO depletion, we used a strain (TU3401), which carries a mutation in *sid-1* (systemic RNA interference defective), a gene that encodes a transmembrane channel for dsRNA, required for systemic RNA interference^46^. To render neurons susceptible to RNAi, a *sid-1* transgene is introduced, driven by a pan-neuronal promoter, *unc-119*^47^. Similarly to the intestine, reduction of *smo-1* expression, specifically in neurons, resulted in shorter lifespan (Fig. 2D). Importantly, overexpression of *smo-1* specifically in the intestine (p_*vha-6*_*smo-1*), led to an extended lifespan (Fig. 2E). On the contrary, neuron-specific *smo-1* overexpression (p_*rab-3*_*smo-1*) did not have any significant effect on the lifespan of the animals (Fig. 2F). This result suggests the importance of balanced SUMO levels in the nervous system. Taken together, these findings indicate that SUMO influences *C. elegans* lifespan through the intestine and the nervous system.

### SUMO regulates the transcriptional activity of SKN-1 and DAF-16

**Figure 3:**
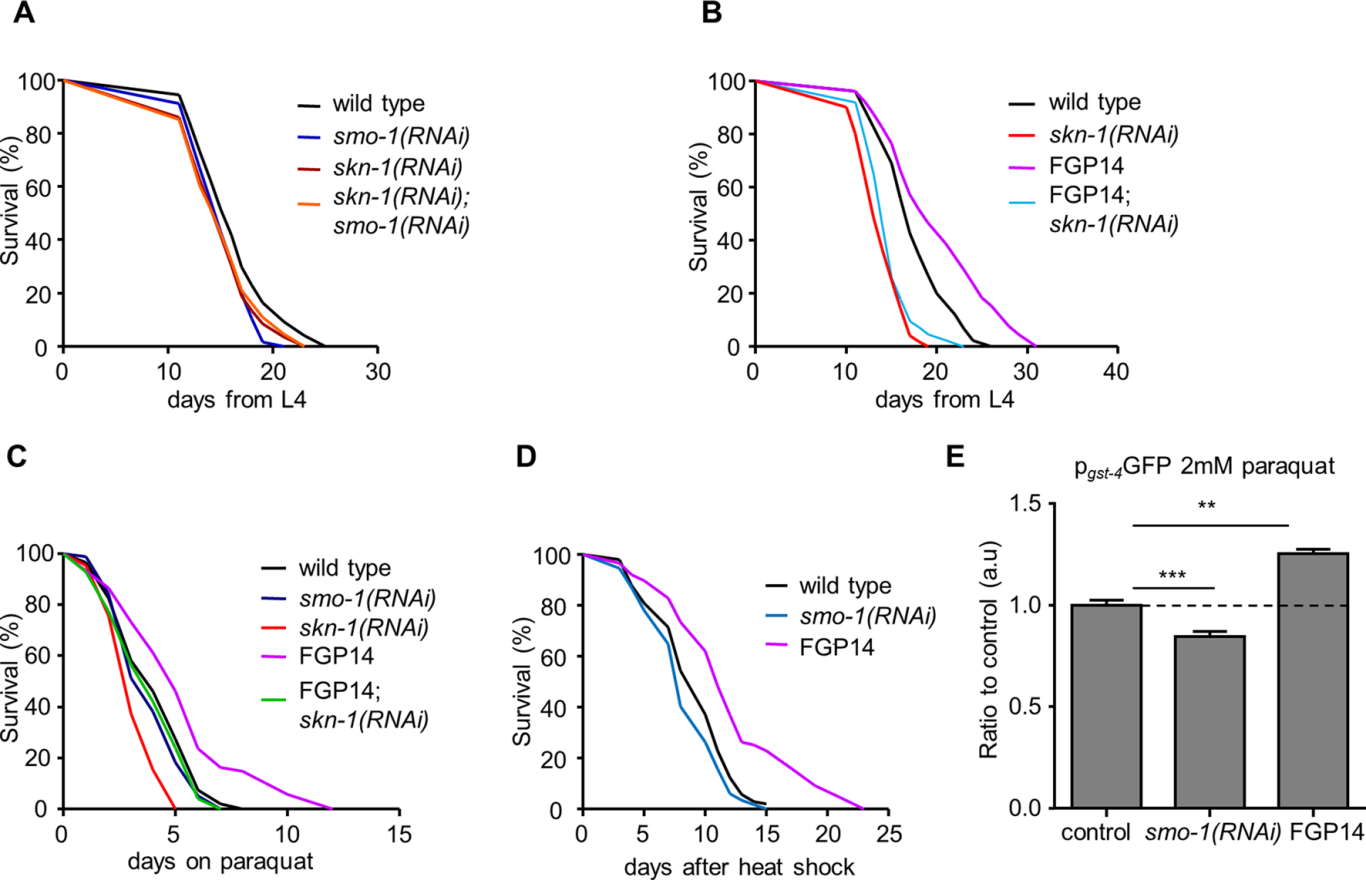
SKN-1 mediates the lifespan influencing effect of SUMO. (**A**) *smo-1(RNAi)* does not further reduce the short lifespan of *skn-1(RNAi)* treated animals. (**B**) *skn-1(RNAi)* shortens the lifespan of the FGP14 strain to the level seen in wild type animals subjected to *skn-1* RNAi. (**C**) SMO-1 overexpressing animals survive longer on 2 mM paraquat, while loss of *smo-1* does not change the survival compared to wild type animals. *skn-1(RNAi)* treated animals have a reduced survival rate on paraquat. (**D**) Heat shock did not change the survival of *smo-1(RNAi)* fed animals while worms overexpressing SMO-1 displayed increased survival rates. The percentage of animals remaining alive is plotted against age. (**E**) FGP14 animals are mounting a stronger oxidative stress response in day 2 animals compared to control, measured by p_*gst-4*_GFP expression. *smo-1(RNAi)* reduced the responsiveness of animals to paraquat. (n = 40, ***p < 0.001, **p < 0.01 unpaired t-test). Error bars, S.E.M. Lifespan assays were carried out at 20 °C. Lifespan values are given in Table S1.

To gain insight relevant to the role of SUMOylation in the regulation of ageing, we examined the effects of SUMO depletion in the context of well-characterized signalling pathways that modulate lifespan in *C. elegans*^5^. HSF-1, SKN-1 and DAF-16 are key stress response transcription factors, promoting longevity under conditions of heat shock, mild oxidative stress and low insulin/IGF1 signalling^8^. Considering, that HSF-1 has been shown to have the potential to be modified by SUMO, both in mammals^48^ and in *C. elegans*^49^, we focused our studies on SKN-1 and DAF-16. We find that *skn-1(RNAi);smo-1(RNAi)* animals display shorter lifespan, similar to animals subjected only to *skn-1* RNAi (Fig. 3A). Importantly, SKN-1 depletion annulled lifespan extension caused by SMO-1 overexpression (Fig. 3B). Thus, SKN-1 mediates the pro-longevity effects of SUMO. Given that the mammalian orthologue of SKN-1, NRF2 is a target for SUMOylation^50,51^, we examined whether SKN-1 is also modified by SUMO conjugation. Detection of SUMO modification in vivo poses multiple challenges (low expression of SKN-1 under normal conditions, low SUMOylation rates of total protein content), and we were unable to observe it in our Western blots (data not shown). To this end, we performed in vitro SUMOylation assays with bacterially expressed 6xHis-MBP tagged and purified SKN-1. We found that SKN-1 is not an optimal SUMO target in vitro (Fig. S2A).

To further investigate the role of SKN-1 in mediating the effects of SUMO on lifespan, we subjected animals to oxidative stress which activates SKN-1^52^. We exposed 6 day old wild type, FGP14 and *smo-1(RNAi)* treated animals to 2 mM paraquat, an inducer of oxidative stress. As expected, *skn-1(RNAi)* treated animals were sensitive to oxidative stress (Fig. 3C). Notably, *smo-1* overexpressing animals were resistant to oxidative stress, while reduction in *smo-1* expression did not alter survival on paraquat (Fig. 3C). We also assayed heat stress resistance upon perturbation of SUMO levels. Similarly to oxidative stress, *smo-1* overexpression increased survival of day 4 animals to acute (2 h) heat shock at 37 °C, whereas, SUMO depletion did not affect heat stress resistance (Fig. 3D). Next, we monitored the expression of *gst-4*, a SKN-1 target gene encoding glutathione S-transferase, levels in day 2 animals on 2 mM paraquat. Using a p_*gst-4*_GFP reporter fusion, we found that knockdown of *smo-1* reduced the expression of *gst-4*, while, overexpression of *smo-1* further increased *gst-4* expression (Fig. 3E). Therefore, under stress conditions, increase of SUMO levels enhances stress resistance by potentiating expression of stress response genes, while depletion of SUMO compromises survival under stress by reducing the capacity to fully mount a response.

Interestingly, under normal conditions, *smo-1* knockdown transiently increased *gst-4* expression during adulthood in day 4 animals, while there was no observable difference by day 8 (Fig. S2B,C). By contrast, *gst-4* expression was not altered upon *smo-1* overexpression during adulthood (day 4 or day 8 animals; Fig. S2B,C). The mRNA levels of *gst-4* mirrored the changes observed with the p_*gst-4*_GFP reporter fusion (Fig. S2D). We find that SKN-1 becomes activated in the absence of the FOXO transcription factor DAF-16 that mediates insulin/IGF1 signalling, in day 4 of adulthood, both in wild type and *smo-1* overexpressing animals (Fig. S2B). SKN-1 activation is diminished by day 8 (Fig. S2C). Hence, although SKN-1 is likely not modified by SUMO in vivo, both are required for normal lifespan. Reduction of SUMO levels triggers activation of SKN-1, while SUMO abundance protects against stress and promotes longevity.

**Figure 4:**
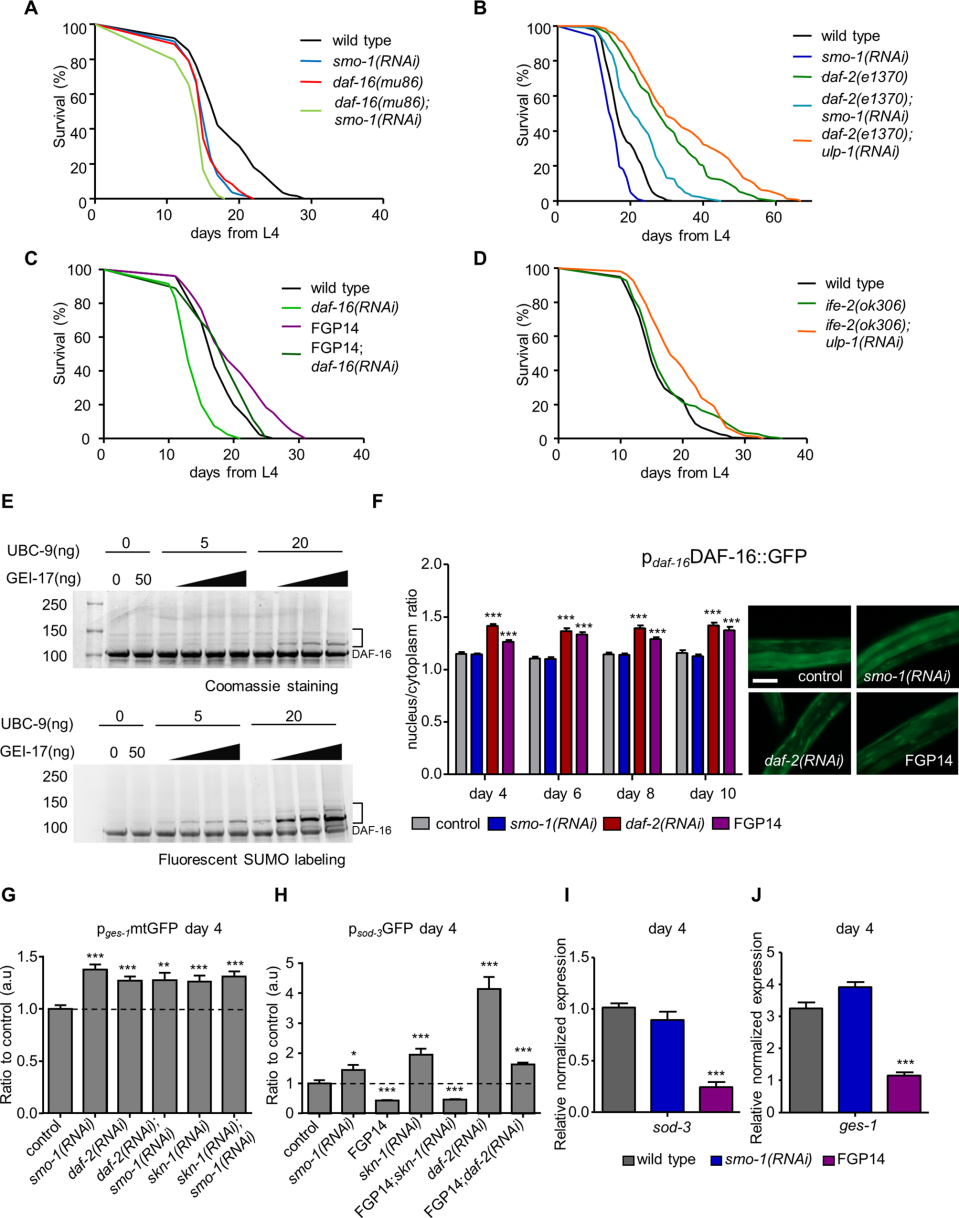
DAF-16 is SUMO modified and SUMO inhibits the transcriptional activity of DAF-16. (**A**) *smo-1(RNAi)* shortens the lifespan of *daf-16(mu86)* animals. (**B**) The long lifespan of *daf-2(e1370)* animals is reduced by *smo-1(RNAi)* and extended by *ulp-1(RNAi)*. (**C**) *daf-16(RNAi)* partly shortens the long lifespan of FGP14 strain. (**D**) *ife-2(ok306)* animals treated with *ulp-1* RNAi have a longer lifespan. (**E**) In vitro DAF-16 SUMOylation assay. Top image shows Coomassie staining, bottom image shows fluorescently labelled SUMO with Alexa-Fluor 680. Brackets indicate the SUMO modified form of DAF-16. (**F**) Nuclear localization of p_*daf-16*_DAF-16::GFP in control, *smo-1(RNAi)* and *smo-1* overexpressing background (FGP14), *daf-2(RNAi)* was used as a positive control, scale bar: 50 μm. Images were acquired using × 40 objective lens (n = 50, ***p < 0.001, unpaired t-test). (**G**) p_*ges-1*_mtGFP expression is elevated upon *smo-1(RNAi)* in wild type, *daf-2(RNAi)* and *skn-1(RNAi)* background in day 4 animals (n = 100, **p < 0.01, ***p < 0.001, unpaired t-test). (**H**) p_*sod-3*_GFP is increased upon knockdown of *smo-1*, *skn-1* or *daf-2*. Overexpression of *smo-1* reduces the expression level of p_*sod-3*_GFP in day 4 animals (n = 100, *p < 0.05, ***p < 0.001, unpaired t-test). (**I**, **J**). The mRNA levels of *sod-3* and *ges-1* are reduced in day 4 animals in the *smo-1* overexpressing strain (***p < 0.001, unpaired t-test). Error bars, S.E.M. Lifespan assays were carried out at 20 °C. Lifespan values are given in Table S1.

In addition to SKN-1, DAF-16 also promotes stress tolerance and longevity^6^. We asked whether SUMO exerts its effects on animal ageing by modulating DAF-16 activity. DAF-16 is activated in long-lived *C. elegans* mutants, carrying lesions in the insulin/IGF1 receptor DAF-2. We find that *smo-1* knockdown shortened the lifespan of DAF-2 deficient animals (Fig. 4A,B). Furthermore, downregulation of *daf-16* abolished lifespan extension caused by *smo-1* overexpression (Fig. 4C). Notably, under lifespan-extending conditions, where DAF-16 is activated (in *daf-2(e1370)* mutants), or protein synthesis is reduced (in *ife-2(ok306)* mutants), downregulation of the SUMO protease *ulp-1* extends lifespan (Fig. 4B,D). These findings indicate that DAF-16 is, in part, mediating the effects of SUMO on lifespan. Thus, we investigated whether DAF-16 is a target for SUMOylation.

We conducted in vitro SUMOylation assays with purified, bacterially expressed and 6xHis-MBP tagged DAF-16. We detected SUMO conjugation to DAF-16 (Fig. 4E top panel, Fig. S3A). To increase the sensitivity of our assay, we added fluorescently labelled SUMO (SUMO-AlexaFluor 680)^35^, which facilitated detection of the SUMO-modified form of DAF-16 (Fig. 4E, bottom panel). We also tested the capacity of the MBP tag to undergo SUMOylation. We found that MBP is not a SUMO target (Fig. S3B). Therefore, DAF-16 is a *bona fide* SUMOylation target.

Under normal conditions, the activity of DAF-16 is repressed by cytoplasmic confinement and degradation^53^. To determine whether changes in SUMO levels modulate DAF-16 activity, we monitored the subcellular localization of the protein by using a full length p_*daf-16*_DAF-16::GFP reporter fusion. Downregulation of *smo-1* did not alter the nuclear/cytoplasm ratio of DAF-16, during adulthood (Fig. 4F). By contrast, *smo-1* overexpression triggered nuclear localization of DAF-16, to an extent similar to that observed in DAF-2 deficient animals (Fig. 4F). Notably, while SUMO depletion increased *daf-16* expression, overexpression of *smo-1* reduced *daf-16* expression (Fig. S3C). Therefore, excess SUMO drives DAF-16 nuclear accumulation. However, subcellular partitioning alone is not enough to predict DAF-16 activity^54^. To gauge the effect of SUMO on the transcriptional activity of DAF-16, we assayed the expression of two typical DAF-16 target genes, *ges-1* (abnormal gut esterase), a gut specific type B carboxylesterase, and *sod-3* (superoxide dismutase), an iron/manganese superoxide dismutase. By using p_*ges-1*_mtGFP and p_*sod-3*_GFP reporter fusions, we found that DAF-16 activity increases in day 4 *smo-1(RNAi)* animals (Fig. 4G,H). This increase persists in older animals (day 8 for p_*ges-1*_mtGFP and day 6 for p_*sod-3*_GFP; Fig. S3D,E). Overexpression of *smo-1* reduced DAF-16 activity (Fig. 4H,J, Fig. S3E). Notably, knockdown of *skn-1* also activated DAF-16 in both wild type and *smo-1* overexpressing animals (Fig. 4G,H, Fig. S3D,E), indicating a compensatory link between DAF-16 and SKN-1.

Since SUMOylation has been implicated in chromatin remodelling^55^, we considered whether *smo-1* overexpression decreased transcription of DAF-16 target genes by inducing chromatin condensation. We found that knockdown of *daf-2* increased *sod-3* expression in *smo-1* overexpressing animals, albeit to a lesser extent, compared to wild type controls (Fig. 3H, Fig. S3E). Taken together, these findings indicate that SUMO represses the transcriptional activity of DAF-16.

### SUMO facilitates mitochondrial homeostasis

**Figure 5:**
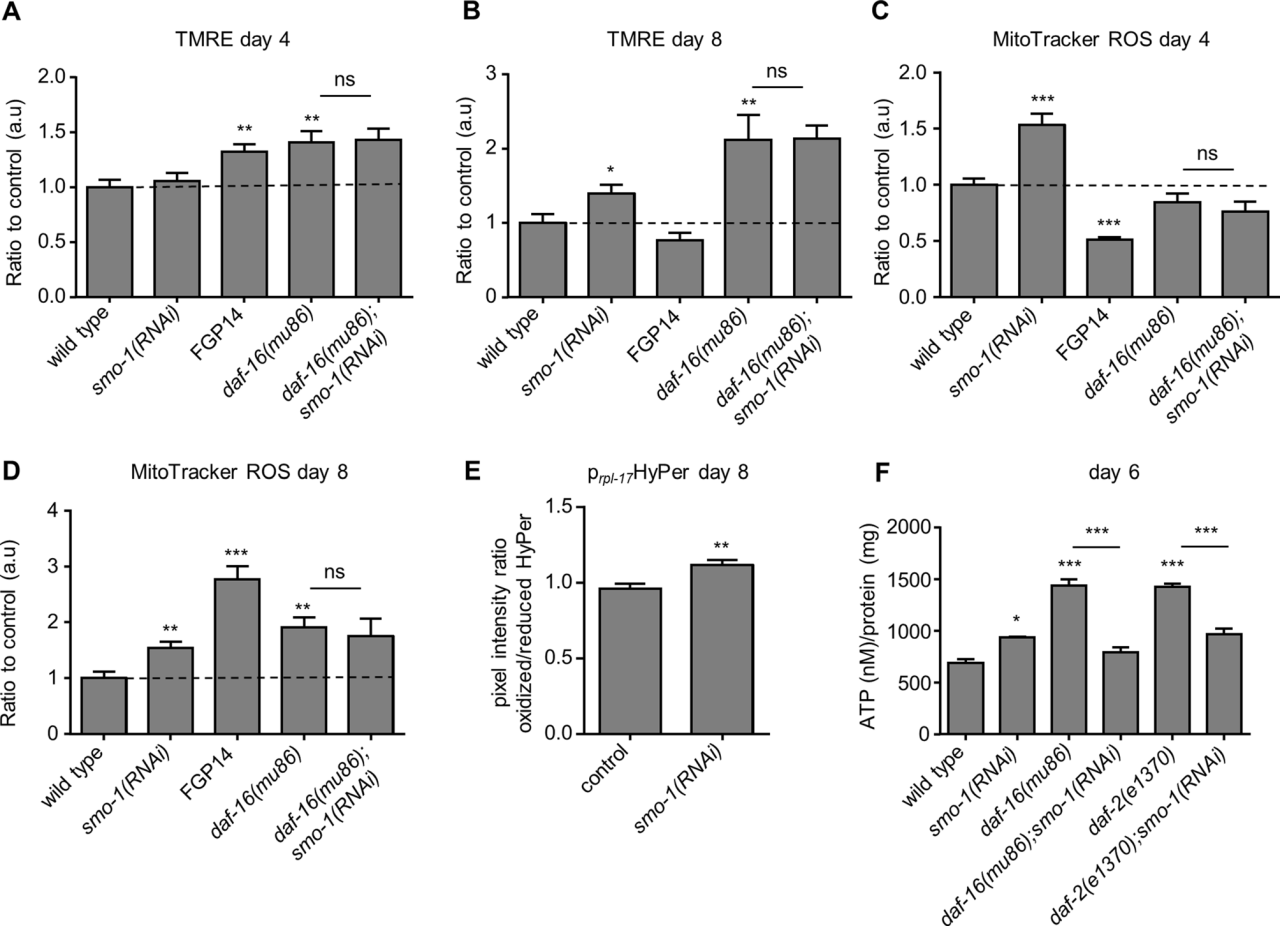
SUMO changes the mitochondrial homeostasis. (**A**, **B**) TMRE staining declines during ageing in wild type but not *smo-1(RNAi)* treated animals, and this change is DAF-16 dependent (n = 100, *p < 0.05, **p < 0.01, ***p < 0.001, unpaired t-test). (**C**, **D**) Mitochondrial ROS production, measured by MitoTracker ROS, is increased when we knockdown *smo-1* and this effect is also DAF-16 dependent (n = 75, *p < 0.05, **p < 0.01, ***p < 0.001, unpaired t-test). (**E**) H_2_O_2_ levels are increased in *smo-1(RNAi)* background, measured by the H_2_O_2_ biosensor, HyPer (n = 30, **p < 0.01, unpaired t-test). (**F**) ATP production is increased upon knockdown of *smo-1* in day 6 wild type animals (*p < 0.05, ***p < 0.001, unpaired t-test). Error bars, S.E.M.

Mitochondrial metabolism is an important determinant of ageing across diverse organisms. We found that SUMOylation levels increase not just in whole worm lysates (Fig. 1A), but also in the mitochondrial fraction (Fig. S4A). To examine whether SUMO impacts lifespan by altering mitochondrial function, we utilized the dye, TMRE (tetramethylrhodamine, ethyl ester), which stains mitochondria according to their membrane potential. Wild type animals display reduced mitochondrial membrane potential during ageing^1^. Nevertheless, knockdown of *smo-1* alleviated age-associated mitochondrial membrane potential decline (Fig. 5A,B). Moreover, animals overexpressing *smo-1* displayed increased TMRE staining early in adulthood (Fig. 5A,B).

Both DAF-16 and SKN-1 are key regulators of the mitochondrial homeostasis^10,56^. We find that knockdown of *smo-1* in genetic backgrounds where DAF-16 is activated, such as *isp-1(qm150)*, *skn-1(RNAi)*, and *daf-2(e1370)* ameliorated mitochondrial membrane potential decline (Fig. S4B,C). Interestingly, mitochondrial membrane potential is elevated in DAF-16 deficient animals and does not increase further upon *smo-1* downregulation (Fig. 5A,B). We also measured mitochondrial ROS production, using the MitoTracker ROS dye. Consistent with the TMRE data; *smo-1* downregulation altered ROS production in all genetic backgrounds tested, except in DAF-16 deficient animals (Fig. 5C,D and S4D,E). Overexpression of *smo-1* increased ROS production during ageing (Fig. 5D). Treatment with the antioxidant NAC (*N*-acetyl cysteine), diminished the extended lifespan of *smo-1* overexpressing animals (Fig. S4F). Additionally, we utilized the p_*rpl-17*_HyPer strain^57^ to determine H_2_O_2_ levels in animals treated with RNAi against *smo-1*. Similarly to MitoTracker ROS staining, aged (8-day old) *smo-1* depleted animals exhibited higher H_2_O_2_ levels compared to control (Fig. 5E). Together, these findings suggest that SUMO represses mitochondrial activity during ageing via DAF-16. As readouts of mitochondrial function, we measured production of ATP and oxygen consumption rates in 4-day old animals. We did not detect significant change upon *smo-1(RNAi)* in wild type or *daf-16(-)* genetic background (Fig. S5A,B). *daf-2(e1370)* animals displayed lower oxygen consumption rates, and *smo-1(RNAi)* did not significantly alter this rate (Fig. S5B). On the contrary, knockdown of *smo-1* resulted in higher ATP production in day 6 wild type animals (Fig. 5F), consistent with the TMRE and MitoTracker ROS staining results (Fig. 5A–D). Intriguingly, SMO-1 depletion reduced ATP levels in *daf-2(e1370)* and *daf-16(mu86)* animals (Fig. 5F), indicating that mitochondrial ROS and ATP production are uncoupled in these genetic backgrounds. To assay mitochondrial content, we used two methods: paraquat (4 mM) treatment followed by TMRE staining and mitochondrial DNA copy number measurement. Administration of paraquat leads to mitochondrial membrane potential dissipation; therefore, accumulation of TMRE reflects the number of mitochondria. Interestingly, downregulation of *smo-1* increased the number of mitochondria during ageing in a DAF-16 dependent manner (Fig. S5C,D); but not mitochondrial DNA copy number (Fig. S5E,F). Thus, mitochondrial function and mass are upregulated upon *smo-1* knockdown during ageing.

**Figure 6:**
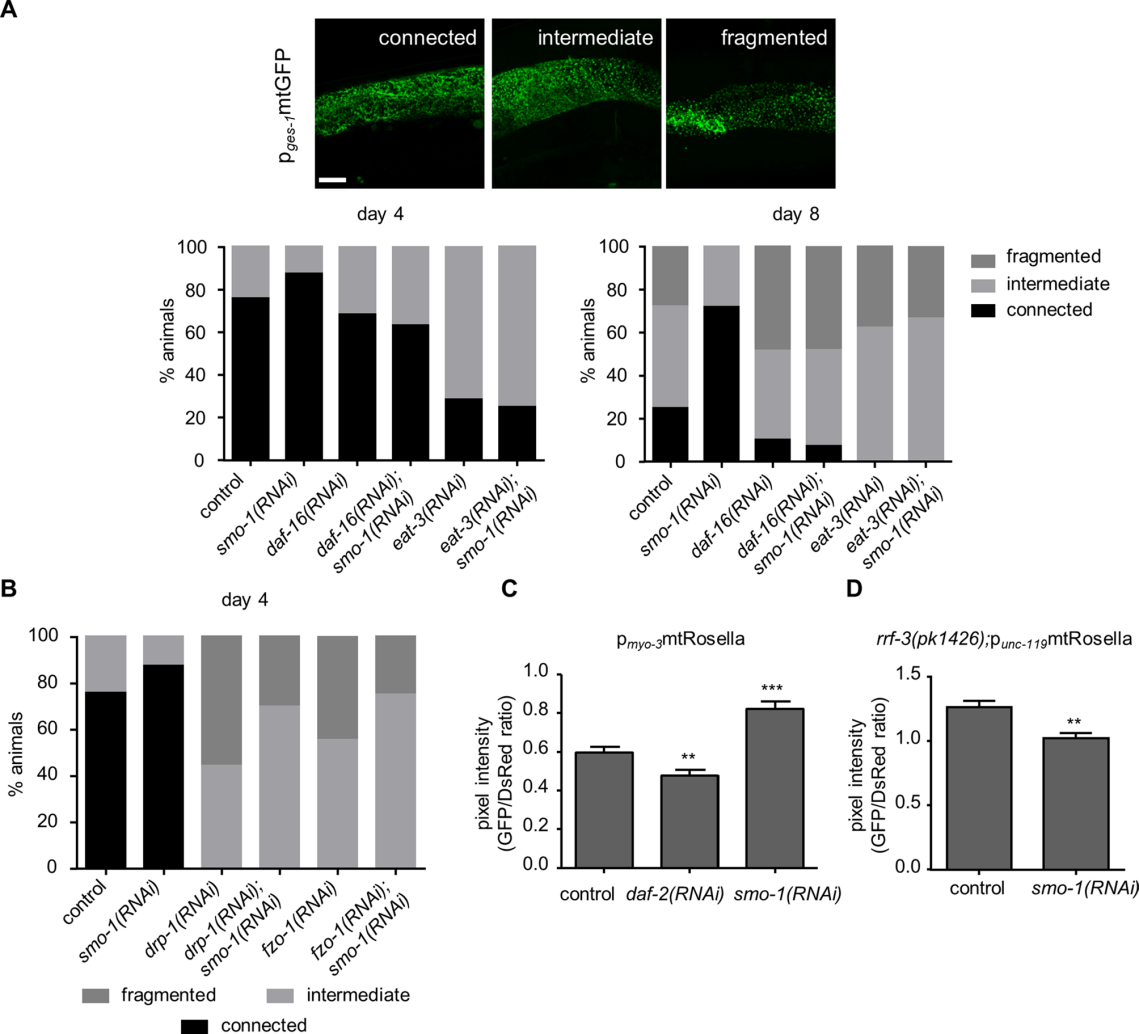
SUMO is required for efficient mitochondrial fission and regulates mitophagy. (**A**) The intestinal mitochondrial network becomes fragmented in day 8 wild type animals, but remains interconnected in *smo-1(RNAi)* treated animals, and the mitochondrial network influencing effect of SMO-1 is DAF-16 and EAT-3 dependent, scale bar: 10 μm. Images were acquired using × 63 objective lens (n = 40). (**B**) In the absence of *drp-1* or *fzo-1* the mitochondrial network undergoes fragmentation, and the loss of *smo-1* rescues this phenotype (n = 15). (**C**) *daf-2(RNAi)* increases, while *smo-1(RNAi)* inhibits mitophagy in muscle cells (n = 40, **p < 0.01, ***p < 0.001, unpaired t-test). (**D**) Depletion of *smo-1* induces neuronal mitophagy (n = 20, **p < 0.01, unpaired t-test). Error bars, S.E.M.

Alongside changes in mitochondrial activity, morphological alterations occur during ageing. The interconnected, tubular mitochondrial network in young animals becomes fragmented as they age^9,10^. We followed mitochondrial morphology during ageing using a mitochondrially localized p_*ges-1*_mtGFP reporter fusion (Fig. 6A). The mitochondrial network displayed extended fragmentation by day 8 of adulthood in wild type, but not in SMO-1 depleted animals (Fig. 6A), indicating a requirement for SUMO for mitochondrial fission. DAF-16 has been implicated in the regulation of mitochondrial morphology^10^. We examined whether SMO-1 modulates mitochondrial morphology through DAF-16. Knockdown of *daf-16* caused mitochondrial network fragmentation in SMO-1 deficient animals, during ageing (Fig. 6A). DAF-16 controls the expression of *eat-3* (eating: abnormal pharyngeal pumping), a gene encoding the homologue of OPA1, a protein required for the fusion of inner mitochondrial membrane^58,59^. EAT-3 deficient animals display a fragile, fragmented intestinal mitochondrial network. Similarly to DAF-16, we find that downregulation of *eat-3* triggered mitochondrial network fragmentation in SMO-1 deficient animals, during ageing (Fig. 6A). These findings indicate that SMO-1 modulates the morphology of mitochondria via DAF-16.

In addition to EAT-3/OPA-1, DRP-1 (dynamin related protein-1) and FZO-1 (Fzo mitochondrial fusion protein related), regulate mitochondrial network remodelling. Notably, DRP1 becomes SUMOylated in mitosis, cell death and ischemia^60–63^. In *C. elegans*, reduced expression of *drp-1* or *fzo-1* leads to mitochondrial network fragmentation^10^ (Fig. 6B). We find that *smo-1* knockdown partially restores mitochondrial network connectivity upon *drp-1* and *fzo-1* downregulation (Fig. 6B). Combined, our observations suggest that SUMO is required for mitochondrial fission and exerts its effect on the mitochondrial fusion/fission machinery via DAF-16.

Previous studies have shown that mitochondrial fission is required for, and precedes mitophagy^64^. Since SUMO impinges on mitochondrial dynamics, we tested whether mitophagy is also affected upon downregulation of *smo-1* expression. To monitor mitophagy, we used a mitochondria targeted, ratiometric Rosella biosensor, expressed in body wall muscle cells^10^. Under mitophagy-inducing, low insulin/IGF1 signalling conditions, in *daf-2* mutants, the ratio of GFP/DsRed fluorescence is reduced, signifying mitophagy induction (Fig. 6C, S6A). By contrast, knockdown of *smo-1* increased the GFP/DsRed ratio, indicating that SUMO depletion inhibits mitophagy (Fig. 6C, S6A). This observation is consistent with the block of mitochondrial fission upon *smo-1* downregulation (Fig. 6B). Additionally, we also assayed mitophagy in the nervous system^65^, in the RNAi sensitive, *rrf-3(pk1426)* mutant background. Surprisingly, reduced *smo-1* expression resulted in the induction of mitophagy in neurons (Fig. 6D, S6B). Thus, SUMO is required to perform mitochondrial fission and mitophagy in a tissue specific manner.

## Discussion

The regulatory role of SUMO in the ageing process remains elusive, up to date. Earlier studies in mice uncovered a progressive increase of protein SUMOylation in brain and plasma, during ageing^29^. Notably, we find a similar increase in the amount of SUMO, during adulthood in *C. elegans*, both in whole worm lysates and in the mitochondrial fraction (Fig. 1A, S4A). Interestingly, reducing the expression of SUMO proteases did not have any lifespan altering effect under normal conditions (Fig. S1B,C). Presumably, the function of these proteases is redundant. Furthermore, under various stress conditions mild heat stress (25 °C), reduced translational rates (*ife-2(-)* mutant background) and low insulin signalling (*daf-2(-)* mutant background) the loss of *ulp-1* resulted in an extended lifespan (Figs. S1D, 4B,D). Therefore, specific SUMO proteases have the potential to regulate longevity upon stress conditions. To define the exact role of each SUMO protease under distinct stress insults is an interesting topic for future investigations. SMO-1 is expressed in all animal tissues and mainly localizes to the nucleus^66^. Accordingly, the most studied functions of SUMO are nucleus-associated^14,67,68^; however, its role outside of the nucleus is emerging^69^, with a focus on the nervous system. Indeed, we find that tightly controlled expression level of *smo-1* is a prerequisite for normal lifespan (Fig. 2). A recent study implicated SUMOylation of the germline RNA binding protein CAR-1 (cytokinesis, apoptosis, RNA-associated), in the regulation of lifespan by insulin signalling. Under low insulin signalling conditions (in *daf-2* mutants), CAR-1 is less likely to be SUMO-modified, and can effectively inhibit *glp-1* expression in the germline, allowing for lifespan extension^28^. Our findings indicate that DAF-16 and SKN-1 mediate the effects of SUMO on ageing, in the soma.

**Figure 7:**
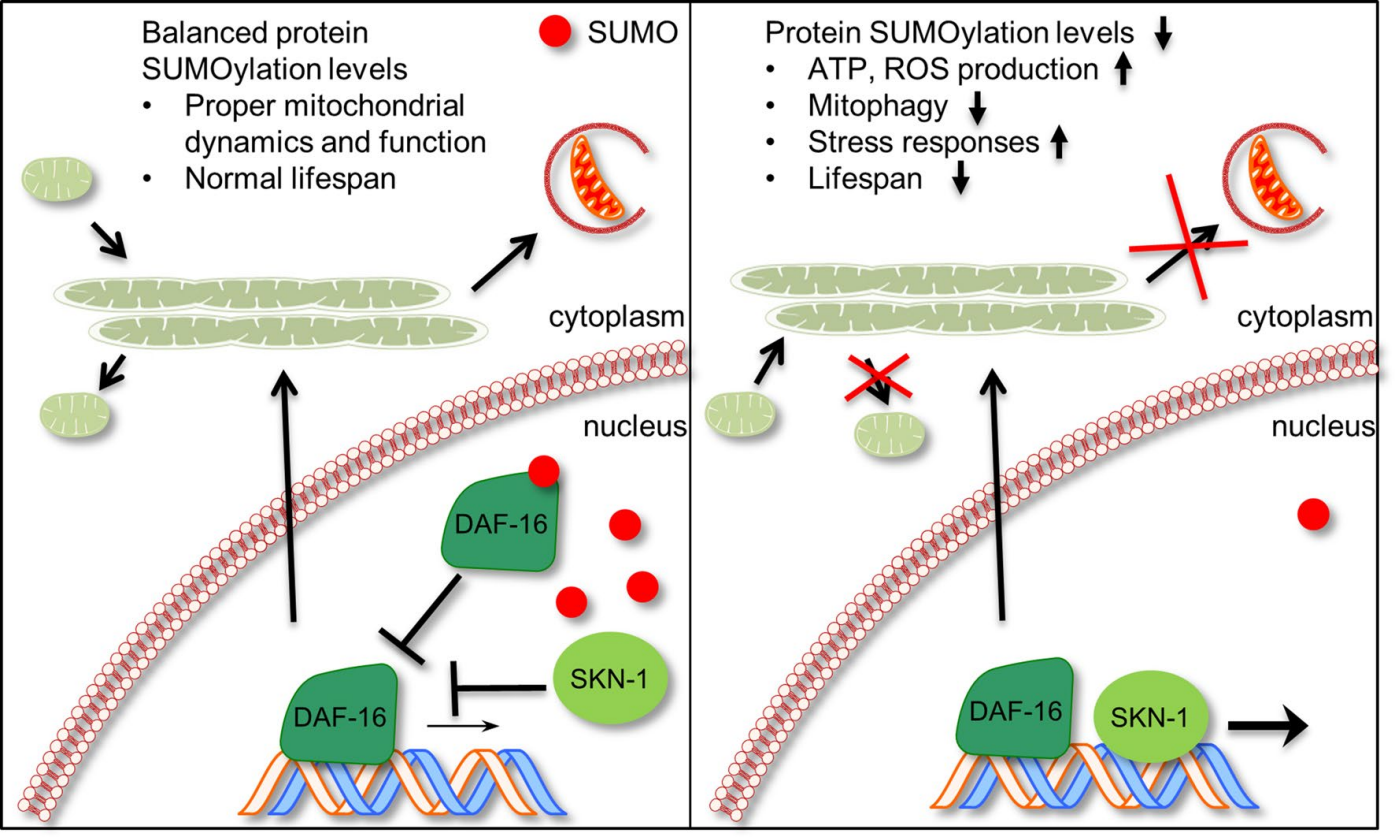
SUMOylation and ageing. Under normal conditions protein SUMOylation is balanced by conjugation and cleavage events. This ensures the tight control of mitochondrial function and dynamics, allowing for a normal lifespan. Depletion of SUMO leads to activation of stress responses, impairment of mitochondrial function and mitophagy, which shortens lifespan. SUMO modulates the activity of the DAF-16 and SKN-1, stress response transcription factors to influence ageing.

SUMOylation of transcription factors is mostly coupled with transcriptional repression^70^. Indeed, we found that DAF-16 is a target for SUMOylation, upon which, its transcriptional activity is quenched (Fig. 4). Under basal conditions this modification could serve to inhibit the unnecessary activation of stress response genes by nuclear localized DAF-16. Indeed, SUMO overexpression leads to the nuclear accumulation of DAF-16 (Fig. 4F); however, without the activation of DAF-16 target genes (Fig. 4G–J). Consequently, the long lifespan of SUMO overexpressing animals is only marginally shortened upon *daf-16* knockdown (Fig. 4C). On the other hand, reduced *skn-1* expression in the SUMO overexpressing background completely abolished lifespan extension in SUMO overexpressing animals (Fig. 3B). These results indicate that the interplay between DAF-16 and SKN-1 is an important determinant of the ageing mechanism. Notably, numerous pathways converge on organismal lifespan^71^; further studies could determine the DAF-16 independent, lifespan extending mechanisms upon SMO-1 overexpression in *C. elegans*. Removal of SUMO results in the upregulation of stress responsive genes, controlled by DAF-16 and SKN-1 (Figs. 4G–J, S2B,D). These findings are consistent with the reported functions of SUMOylation in the regulation of stress responses (e.g. DNA damage response, ER stress, heat shock), which are indispensable for cellular survival^72^. Furthermore, a recent study indicates that activation of SKN-1 negatively regulates DAF-16, which is in agreement with our data showing the increased expression of DAF-16 target genes upon *skn-1(RNAi)*^73^ (Fig. 4G,H). It would be interesting to analyse the role of SUMO in this interaction. Our results hint towards the possibility of the requirement of SUMO modification of DAF-16 for the successful SKN-1 repression. Thus, SUMO depletion compromises cellular homeostasis, and may, additionally, generate a state of cellular stress that contributes to early organismal death (Fig. 7).

SUMOylation has also been linked to mRNA translation regulation^74^, and chromatin organization^55^. Notably, hyposumoylation of chromatin has been associated with pluripotency reprogramming and enhanced lineage trans-differentiation^75^. We considered whether the changes in transgene expression upon SUMO depletion, or overexpression, in *C. elegans* are an indirect consequence of alterations in chromatin structure, and/or mRNA translation. We observed that downregulation of *daf-2* is sufficient to restore expression of *sod-3*, a DAF-16 target gene, which is significantly reduced upon SUMO overexpression (Fig. 4H), indicating that the chromatin is still accessible under this condition. Furthermore, expression of *gst-4*, a SKN-1 target gene, is not affected by SUMO overexpression (Fig. S2B–D) and increases following paraquat treatment (Fig. 3E). By contrast, SUMO deficiency results in the upregulation of both *sod-3*, and *gst-4* expression (Figs. 4H, S2B). Therefore, loss of SUMO engages stress response pathways, involving both DAF-16 and SKN-1 transcription factors (Fig. 7).

SUMOylation has been linked to mitochondrial biogenesis and network remodelling through PGC-1α and Drp1 in mammals^63,76,77^. Moreover, SUMO modification of ATFS-1 and DVE-1 play a regulatory role in the process of mitochondrial unfolded protein response (^mt^UPR)^78^. Here, we examined the involvement of SUMO in mitochondrial homeostasis. We observed an increase in mitochondrial content, but not in mitochondrial DNA copy number upon depletion of SUMO in *C. elegans* (Fig. S5C–F). Additionally, ATP and ROS production increases in aged animals, indicating elevated mitochondrial activity (Fig. 5C–F). Moreover, we found that SUMO promotes mitochondrial fragmentation during ageing (Fig. 6A,B) and it is also required for mitochondrial turnover via mitophagy in muscle cells (Fig. 6C). Intriguingly, SUMO blocks the process of mitophagy in the nervous system (Fig. 6D). Admittedly, increased mitophagy has been shown to have detrimental consequences on the homeostasis of the cell^79,80^. The tissue specific effects of SUMO on mitophagy merits further research. Notably, while in long-lived mutants, mitochondria display elongated morphology, coupled with reduced ROS production^58^, we find that short-lived, SUMO-depleted animals also feature elongated mitochondrial network, but elevated generation of ROS. This discrepancy indicates that mitochondrial turnover is differentially affected in long-lived, and short-lived, SUMO deficient mutants. In long-lived animals a healthy interconnected mitochondrial network is maintained by increased mitophagy, which moderates ROS production^10^. Instead, SMO-1 knockdown interferes with mitochondrial fission and fusion, which in turn impairs mitophagy, resulting in accumulation of mitochondrial damage, higher ROS levels, and shorter lifespan. Our observations suggest that SUMO decreases mitochondrial function and promotes mitochondrial fission during ageing in *C. elegans* (Fig. 7). These findings are consistent with recent studies suggesting that impairment of mitochondrial dynamics contributes to the decline of mitochondrial function during ageing and the onset of age-related diseases^81^.

Collectively, our findings indicate that SUMO influences the ageing process by modulating the transcriptional activity of the stress response transcription factors, DAF-16 and SKN-1 (Fig. 7). Admittedly, these are not the only transcription factors that could be affected by the depletion of SUMO. Further research is required to fully map the molecular changes in response to altered SUMO levels. Notably, recent studies have implicated SUMO in senescent decline. SUMOylation of p53 causes cellular senescence^82^, while deSUMOylation of Bmi1, a polycomb repressive complex member, is likewise, required for senescence^83^. Moreover, the SUMO E2 enzyme, Ubc9, regulates senescence by relocation of SUMOylated proteins to PML nuclear bodies^84^. Thus balanced protein SUMOylation is critical for stress resistance and survival. The ageing process disrupts SUMOylation balance while manipulations that fine-tune protein SUMOylation promote longevity.

Here, we demonstrate that the abundance of SUMO regulates lifespan. Reduced SUMO levels shorten lifespan, while increased *smo-1* expression results in extended lifespan (Figs. 2, 7). SUMO influences ageing mainly through DAF-16 and SKN-1 (Figs. 3A,B, 4A,B), in a tissue specific manner, through the intestine and the nervous system (Fig. 2). We further show that DAF-16 is a target for SUMOylation, and that SUMO attachment represses the transcriptional activity of DAF-16 (Figs. 4, 7). We propose that under normal conditions, modification of DAF-16 by SUMO could prevent uncontrolled activation of nuclearly localized DAF-16 (Fig. 7). Accordingly, overabundance of SUMO leads to strong DAF-16 inhibition (Fig. 4H–J). Nonetheless, SUMO overexpressing animals exhibit long lifespan, which is dependent on SKN-1 (Fig. 3B) and in a lesser extent on DAF-16 (Fig. 4C). In addition, SUMO plays a critical role in the maintenance of mitochondrial homeostasis. Altering SUMO levels affects mitochondrial ATP and ROS production (Fig. 5), as well as, mitochondrial dynamics and clearance (Figs. 6, 7). Combined, our findings indicate that balanced protein SUMOylation is a prerequisite for healthy animal ageing.

## Supplementary information

Supplementary Information.
