# Supplementary material for: SUMO promotes longevity and maintains mitochondrial homeostasis during ageing in Caenorhabditis elegans
Source: Sci Rep. 2020 Sep 23;10:15513. doi: 10.1038/s41598-020-72637-9 (PMC7511317; doi:10.1038/s41598-020-72637-9)
Supplement: Supplementary file 1 — Supplementary Information. [file 41598_2020_72637_MOESM1_ESM.docx]

**SUMO promotes longevity and maintains mitochondrial homeostasis during ageing in *Caenorhabditis elegans***

Andrea Princz^1,2^, Federico Pelisch^3^ and Nektarios Tavernarakis^1,4^

1 Institute of Molecular Biology and Biotechnology, Foundation for Research and Technology – Hellas, Heraklion, Greece

2 Department of Biology, University of Crete, Heraklion, Greece

3 Centre for Gene Regulation and Expression, Sir James Black Centre, School of Life Sciences, University of Dundee, Dundee, Scotland, UK

4 Department of Basic Sciences, Faculty of Medicine, University of Crete, Heraklion, Greece

**Supplemental Information**

Supplemental Figures S1-S6

Supplemental Table S1

**Supplemental figures & legends**


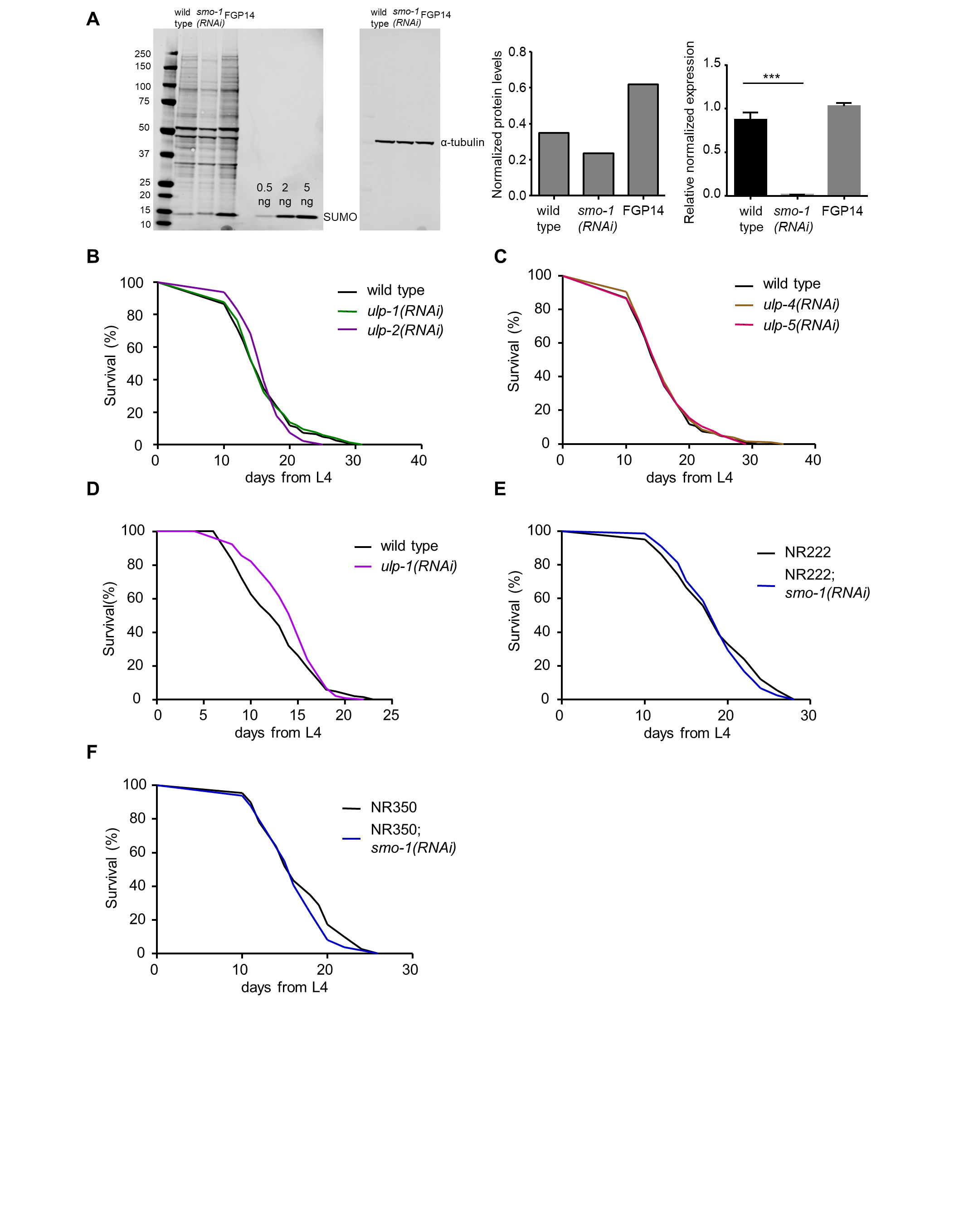


**Figure S1. SUMO proteases do not influence lifespan in wild type animals.**

A Left panel: Western blot showing SUMO protein levels in wild type, *smo-1(RNAi)* and *smo-1* overexpressing (FGP14) background. Protein levels were normalized to α-tubulin. N=1.
Right panel: mRNA levels are diminished under *smo-1(RNAi)* conditions (***p<0.001, unpaired t-test). Error bars, S.E.M.

B-C Removal of SUMO proteases (*ulp-1-5*) does not have an effect on wild type lifespan at 20°C.

D Reduced *ulp-1* expression extends the lifespan of wild type animals at 25°C.

E-F Muscle (NR350) or hypodermis (NR222) specific knock-down of *smo-1* does not have an effect on lifespan. Lifespan assays were carried out at 20°C. Lifespan values are given in Table S1.


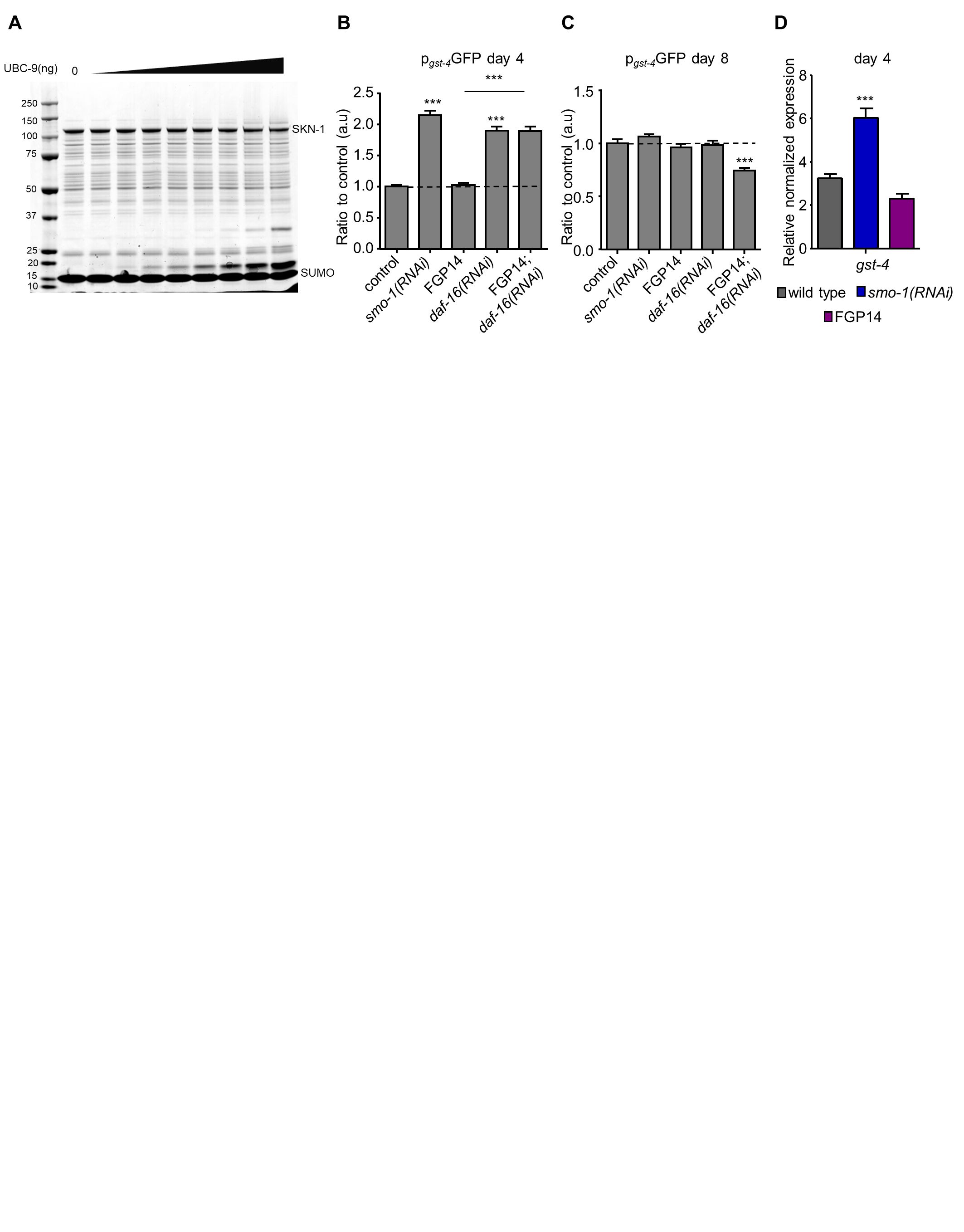


**Figure S2. SKN-1 is not a direct SUMO target.**

A *In vitro* SUMOylation assay using MBP tagged SKN-1.

B-C p*_gst-4_*GFP expression is increased upon *smo-1(RNAi)* and *daf-16(RNAi)* but does not change when *smo-1* is overexpressed in day 4 animals and this change is not observable in day 8 animals (n=100, ***p<0.001, one-way ANOVA).

D The mRNA levels of *gst-4* are increased when we knock-down *smo-1*, but there is no change upon *smo-1* overexpression in day 4 animals (***p<0.001, unpaired t-test). Error bars, S.E.M.


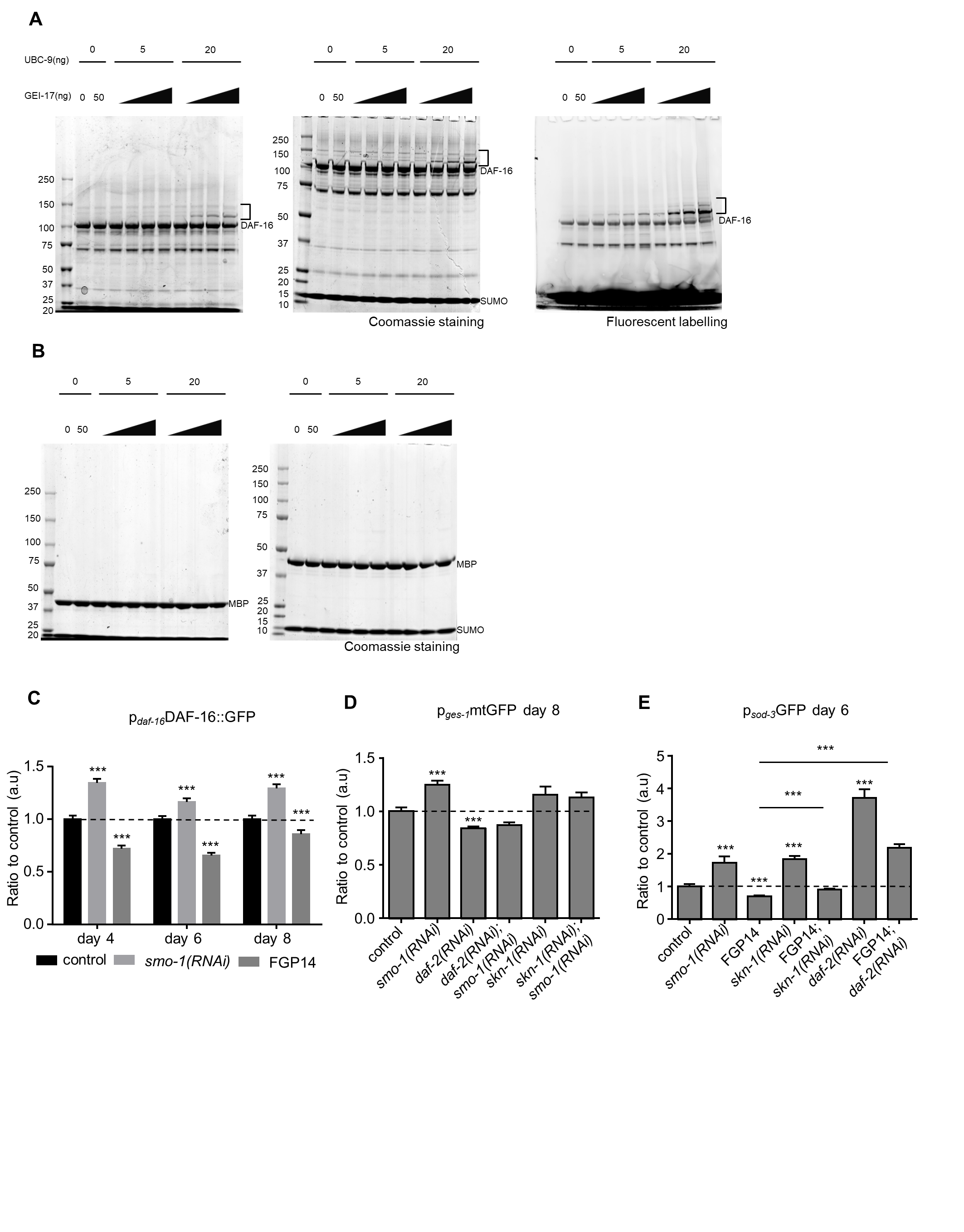


**Figure S3. SUMOylation of DAF-16 modulates stress responses.**

A *In vitro* SUMOylation assay with MBP tagged DAF-16. Brackets indicate the SUMO modified form of DAF-16. Left panel: 3-8% Tris-Acetate gel, Coomassie staining; middle panel: 4-12% Bis-Tris gel, Coomassie staining; right panel: fluorescent labelling of the 3-8% Tris-Acetate gel.

B *In vitro* SUMOylation assay with the MBP tag alone. Left panel: 3-8% Tris-Acetate gel, right panel: 4-12% Bis-Tris gel.

C Decreasing *smo-1* expression increases p*_daf-16_*DAF-16::GFP expression, while overexpression of *smo-1* reduces it (n=100, ***p<0.001, unpaired t-test).

D p*_ges-1_*mtGFP expression is increased on *smo-1(RNAi)* in wild type background in day 8 animals (n=100, ***p<0.001, unpaired t-test).

E p*_sod-3_*GFP expression is up-regulated upon *smo-1(RNAi)*, *skn-1(RNAi)* and *daf-2(RNAi)* in day 6 animals. The overexpression of *smo-1* decreases the expression of *sod-3* (n=75, ***p<0.001, unpaired t-test). Error bars, S.E.M.


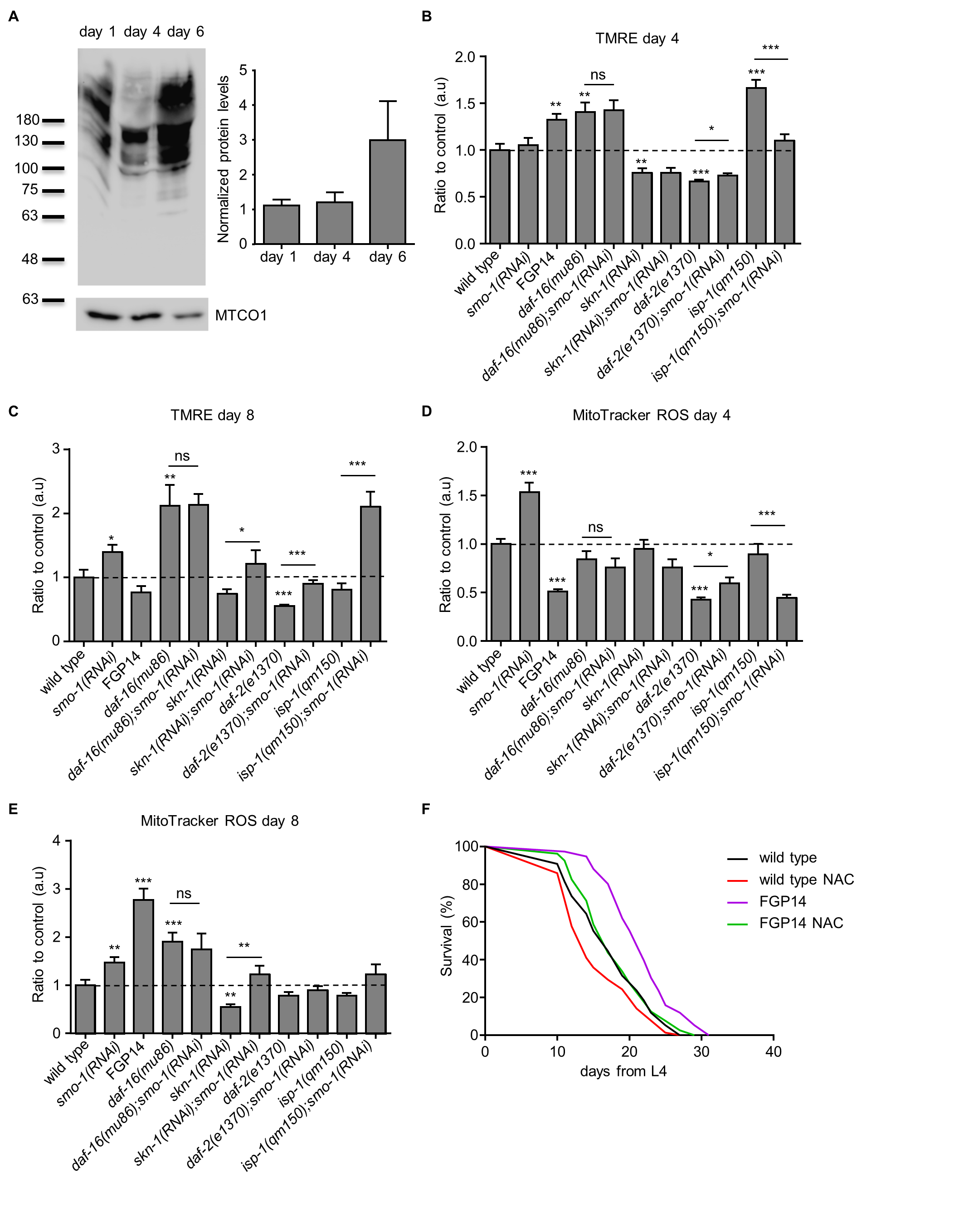


**Figure S4. SUMO levels increase in the mitochondrial fraction and SUMO alters mitochondrial function.**

A Western blot analysis of SUMOylated proteins in the mitochondrial fraction in day 1, 4 and 6 wild type animals (N=4). Protein levels were normalized to MTCO1.

B-C TMRE staining declines during ageing in wild type but not *smo-1(RNAi)* treated animals, and this change is DAF-16 dependent (n=100, *p<0.05, **p<0.01, ***p<0.001, unpaired t-test).

D-E Mitochondrial ROS production, measured by MitoTracker ROS, is increased when we knockdown *smo-1* and this effect is also DAF-16 dependent (n=75, *p<0.05, **p<0.01, ***p<0.001, unpaired t-test). Error bars, S.E.M.

F Treatment with the antioxidant NAC (N-acetyl cyteine) abrogates the long lifespan of *smo-1* overexpressing animals. Lifespan assays were carried out at 20°C. Lifespan values are given in Table S1.


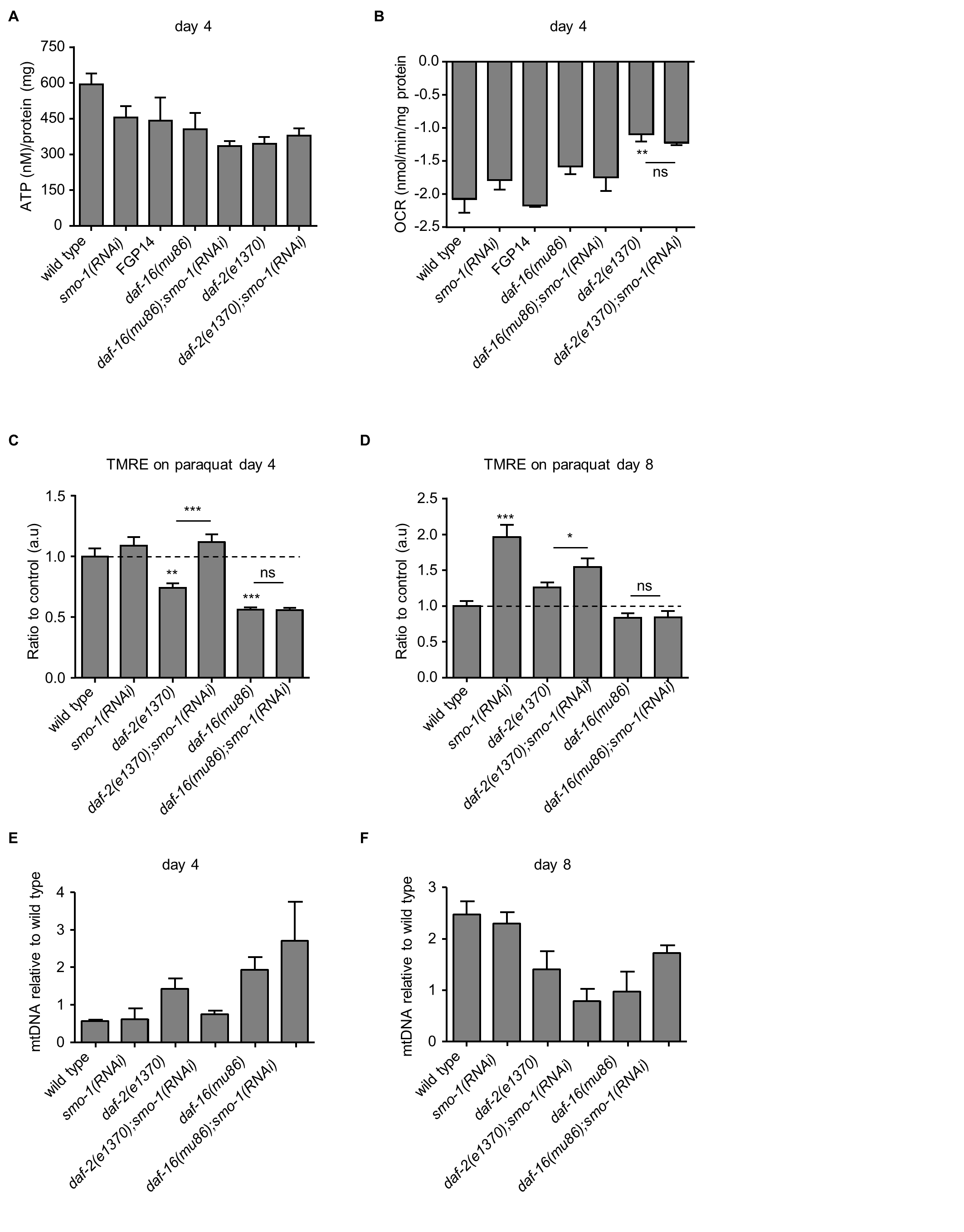


**Figure S5. SUMO regulates mitochondrial content during ageing.**

A ATP levels do not change in 4-day old animals upon *smo-1* knockdown. Error bars, S.E.M.

B Oxygen consumption rates are not affected by *smo-1* depletion at day 4, while *daf-2(e1370)* animals exhibit lower oxygen consumption (**p<0.01).

C-D Mitochondrial content is increased during ageing upon *smo-1(RNAi)* in a DAF-16 dependent manner, measured by subsequent paraquat treatment and TMRE staining (n=50, *p<0.05, **p<0.01, ***p<0.001, one-way ANOVA).

E-F Mitochondrial DNA copy number, normalized to genomic DNA. *smo-1(RNAi)* treatment does not affect the mitochondrial DNA copy number in wild type, *daf-2(e1370)* and *daf-16(mu86)* background in day 4 or day 8 animals. Error bars, S.E.M.


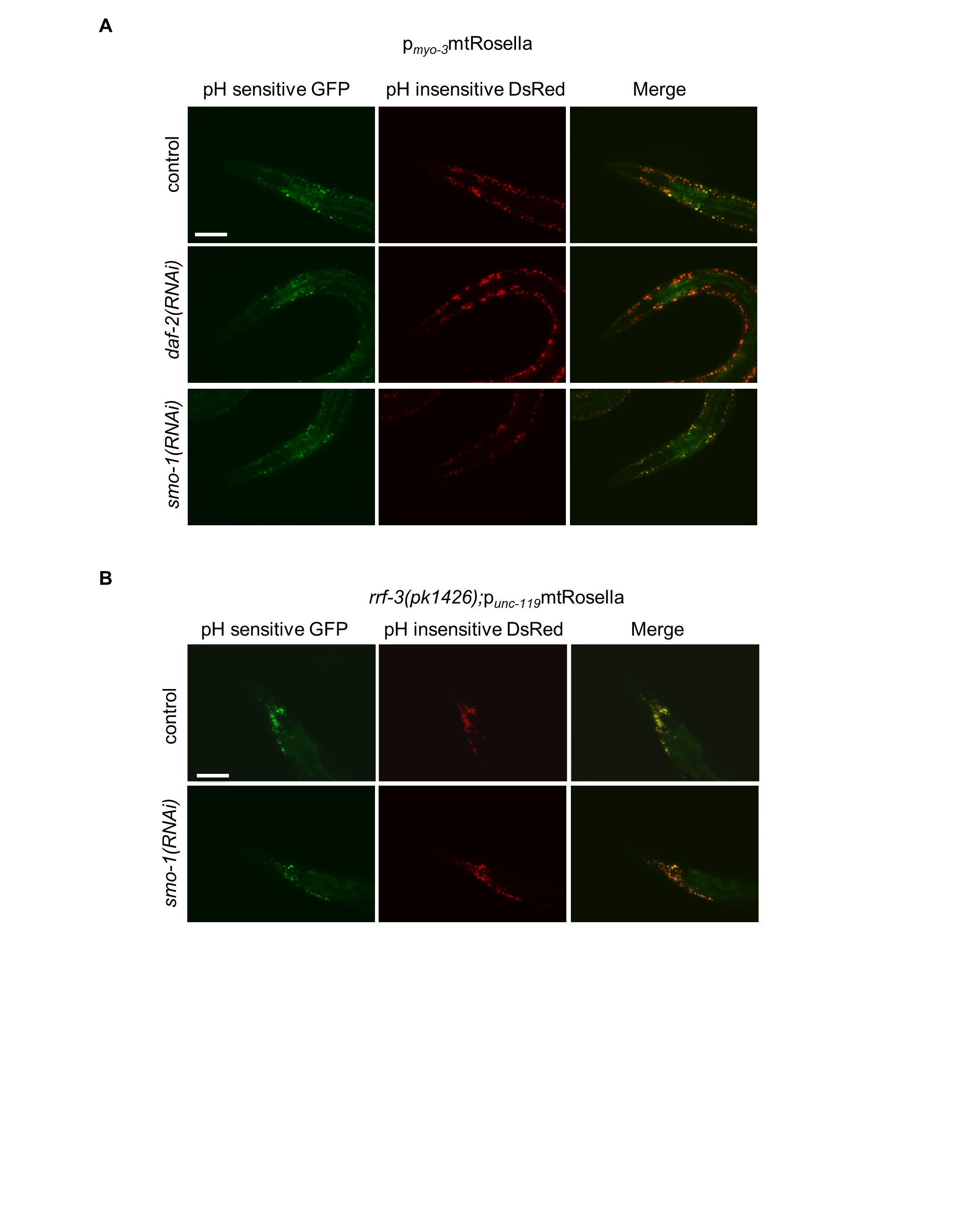


**Figure S6. SUMO influences mitophagy in muscles and neurons.**

A Animals expressing mitochondria targeted Rosella biosensor in body wall muscle cells. *daf-2(RNAi)* increases, while *smo-1(RNAi)* inhibits mitophagy, scale bar: 100 μm. Images were aquired using x20 objective lens.

B Mitochondria targeted Rosella biosensor, expressed in neurons. Knockdown of *smo-1* induces neuronal mitophagy, scale bar: 100 μm. Images were aquired using x20 objective lens.

**Table S1. Lifespan data**

Unless noted otherwise, all aging experiments were performed on plates seeded with HT115(DE3) *E. coli* bacteria, carrying the indicated RNAi plasmid constructs. (Maximum lifespan shown is the median lifespan of the longest-lived 10% of the animals assayed. *P* values were calculated using the log-rank test, as described in Methods.)

| Strain | Median lifespan | Deaths | Censored events | Maximum lifespan | P value | Comparison | Relevant figure |
| --- | --- | --- | --- | --- | --- | --- | --- |
| Experiment #1 | | | | | | | |
| wild type | 16 | 133 | 11 | 27.5 |  |  |  |
| *smo-1(RNAi)* | 15 | 110 | 40 | 20 | <0.0001 | compared to wild type |  |
| Experiment #2 | | | | | | | |
| wild type | 15 | 187 | 9 | 26.5 |  |  |  |
| *smo-1(RNAi)* | 14 | 211 | 10 | 23 | 0.0231 | compared to wild type |  |
| *ulp-1(RNAi)* | 15 | 197 | 8 | 27 | 0.6803 | compared to wild type | graph shown in Figure S1B |
| *ulp-4(RNAi)* | 15 | 200 | 7 | 28.5 | 0.5087 | compared to wild type | graph shown in Figure S1C |
| *ulp-5(RNAi)* | 15 | 227 | 8 | 26.5 | 0.7478 | compared to wild type | graph shown in Figure S1C |
| *daf-16(mu86)* | 14 | 193 | 6 | 22.5 |  |  |  |
| *daf-16(mu86);smo-1(RNAi)* | 14 | 219 | 8 | 22.5 | 0.7567 | compared to *daf-16(mu86)* |  |
| *daf-2(e1370)* | 37 | 200 | 5 | 55.5 |  |  |  |
| *daf-2(e1370);smo-1(RNAi)* | 29 | 204 | 8 | 38 | <0.0001 | compared to *daf-2(e1370)* |  |
| *daf-2(e1370);ulp-1(RNAi)* | 37 | 209 | 11 | 64 | 0.4889 | compared to *daf-2(e1370)* |  |
| VP303 | 20 | 210 | 15 | 25 |  |  |  |
| VP303;*smo-1(RNAi)* | 18 | 227 | 10 | 23.5 | 0.0031 | compared to VP303 |  |
| NR222 | 18 | 225 | 8 | 23.5 |  |  |  |
| NR222;*smo-1(RNAi)* | 18 | 227 | 13 | 23.5 | 0.163 | compared to NR222 |  |
| NR350 | 16 | 155 | 10 | 21.5 |  |  |  |
| NR350;*smo-1(RNAi)* | 16 | 152 | 12 | 21.5 | 0.9375 | compared to NR350 |  |
| Experiment #3 | | | | | | | |
| wild type | 15 | 169 | 3 | 26 |  |  |  |
| *ulp-4(RNAi)* | 17.5 | 190 | 6 | 26 | 0.0031 | compared to wild type |  |
| Experiment #4 | | | | | | | |
| wild type | 17 | 220 | 8 | 27.5 |  |  |  |
| *smo-1(RNAi)* | 15 | 226 | 14 | 20.5 | <0.0001 | compared to wild type |  |
| *ulp-1(RNAi)* | 17 | 236 | 9 | 26.5 | 0.2153 | compared to wild type |  |
| *ulp-4(RNAi)* | 17 | 177 | 8 | 27 | 0.8859 | compared to wild type |  |
| VP303 | 20 | 216 | 12 | 27 |  |  |  |
| VP303;*smo-1(RNAi)* | 20 | 183 | 8 | 25 | 0.009 | compared to VP303 |  |
| NR222 | 18 | 230 | 9 | 28 |  |  |  |
| NR222;*smo-1(RNAi)* | 19 | 270 | 15 | 26.5 | 0.2915 | compared to NR222 |  |
| NR350 | 16 | 199 | 12 | 27 |  |  |  |
| NR350;*smo-1(RNAi)* | 16 | 168 | 10 | 22.5 | 0.0235 | compared to NR350 |  |
| Experiment #5 | | | | | | | |
| wild type | 17 | 153 | 3 | 24 |  |  |  |
| *smo-1(RNAi)* | 13 | 174 | 7 | 21 | <0.0001 | compared to wild type |  |
| TU3401 | 14 | 211 | 3 | 22 |  |  |  |
| TU3401;*smo-1(RNAi)* | 13 | 206 | 5 | 18.5 | <0.0001 | compared to TU3401 |  |
| VP303 | 17 | 102 | 5 | 26 |  |  |  |
| VP303;*smo-1(RNAi)* | 16 | 130 | 5 | 23 | 0.0038 | compared to VP303 | graph shown in Figure 2C |
| NR222 | 18 | 183 | 7 | 27 |  |  |  |
| NR222;*smo-1(RNAi)* | 18 | 211 | 10 | 26 | 0.3457 | compared to NR222 | graph shown in Figure S1E |
| NR350 | 16 | 69 | 8 | 24 |  |  |  |
| NR350;*smo-1(RNAi)* | 16 | 49 | 5 | 23 | 0.3075 | compared to NR350 | graph shown in Figure S1F |
| Experiment #6 | | | | | | | |
| wild type | 16 | 153 | 7 | 23 |  |  |  |
| *smo-1(RNAi)* | 14 | 169 | 12 | 19 | <0.0001 | compared to wild type |  |
| *ulp-1(RNAi)* | 16 | 156 | 6 | 24 | 0.2972 | compared to wild type |  |
| *ulp-2(RNAi)* | 16 | 177 | 8 | 22.5 | 0.999 | compared to wild type | graph shown in Figure S1B |
| *skn-1(RNAi)* | 14 | 193 | 9 | 19 | <0.0001 | compared to wild type |  |
| *skn-1(RNAi);smo-1(RNAi)* | 15 | 178 | 11 | 18.5 | 0.0056 | compared to wild type |  |
| Experiment #7 | | | | | | | |
| wild type | 18 | 74 | 12 | 23 |  |  |  |
| *ulp-2(RNAi)* | 16 | 61 | 10 | 23 | 0.5969 | compared to wild type |  |
| FGP14 | 22 | 186 | 12 | 29 | <0.0001 | compared to wild type |  |
| VP303 | 16 | 95 | 4 | 24 |  |  |  |
| VP303;*smo-1(RNAi)* | 16 | 109 | 3 | 21 | 16 | compared to VP303 |  |
| Experiment #8 | | | | | | | |
| wild type | 16 | 165 | 6 | 23 |  |  |  |
| *smo-1(RNAi)* | 15 | 192 | 8 | 20 | 0.0001 | compared to wild type | graph shown in Figure 3A |
| *skn-1(RNAi)* | 15 | 201 | 8 | 21 | 0.001 | compared to wild type | graph shown in Figure 3A |
| *skn-1(RNAi);smo-1(RNAi)* | 15 | 238 | 12 | 22 | 0.0031 | compared to wild type | graph shown in Figure 3A |
| FGP14 | 19 | 131 | 8 | 29 | <0.0001 | compared to wild type | graph shown in Figure 3B |
| FGP14;*daf-16(RNAi)* | 18.5 | 100 | 15 | 24 | 0.0007 | compared to FGP14 | graph shown in Figure 4C |
| FGP14;*skn-1(RNAi)* | 14 | 176 | 13 | 20.5 | <0.0001 | compared to FGP14 | graph shown in Figure 3B |
| *daf-2(e1370)* | 43 | 130 | 12 | 54.5 |  |  |  |
| *daf-2(e1370);smo-1(RNAi)* | 30 | 163 | 5 | 38 | <0.0001 | compared to *daf-2(e1370)* |  |
| Experiment #9 | | | | | | | |
| wild type | 17 | 203 | 2 | 27 |  |  |  |
| *smo-1(RNAi)* | 15 | 193 | 12 | 21 | <0.0001 | compared to wild type |  |
| *ulp-1(RNAi)* | 17 | 196 | 2 | 28 | 0.6387 | compared to wild type |  |
| TU3401 | 16 | 206 | 5 | 22.5 |  |  |  |
| TU3401;*smo-1(RNAi)* | 16 | 161 | 14 | 19.5 | 0.0018 | compared to TU3401 |  |
| *ife-2(ok306)* | 16 | 214 | 5 | 36 |  |  |  |
| *ife-2(ok306);ulp-1(RNAi)* | 18 | 162 | 2 | 33 | 0.0001 | compared to *ife-2(ok306)* | graph shown in Figure 4D |
| Experiment #10 | | | | | | | |
| wild type | 17 | 228 | 1 | 29 |  |  |  |
| *smo-1(RNAi)* | 15 | 214 | 10 | 22 | <0.0001 | compared to wild type |  |
| *ulp-1(RNAi)* | 18 | 212 | 2 | 26 | 0.5279 | compared to wild type |  |
| *daf-2(e1370)* | 37 | 203 | 12 | 64 |  |  |  |
| *daf-2(e1370);smo-1(RNAi)* | 27 | 220 | 3 | 41 | <0.0001 | compared to *daf-2(e1370)* |  |
| *daf-2(e1370);ulp-1(RNAi)* | 38 | 168 | 18 | 79 | 0.1617 | compared to *daf-2(e1370)* |  |
| Experiment #11 | | | | | | | |
| wild type | 20 | 213 | 5 | 30.5 |  |  |  |
| *smo-1(RNAi)* | 15 | 195 | 4 | 25.5 | <0.0001 | compared to wild type |  |
| TU3401 | 17 | 227 | 3 | 25.5 |  |  |  |
| TU3401;*smo-1(RNAi)* | 16 | 231 | 3 | 21 | <0.0001 | compared to TU3401 | graph shown in Figure 2D |
| *ife-2(ok306)* | 21 | 234 | 6 | 42 |  |  |  |
| *ife-2(ok306);ulp-1(RNAi)* | 20 | 252 | 4 | 42 | 0.0198 | compared to *ife-2(ok306)* |  |
| Experiment #12 | | | | | | | |
| wild type | 18 | 202 | 6 | 29.5 |  |  |  |
| *smo-1(RNAi)* | 18 | 255 | 13 | 24 | <0.0001 | compared to wild type |  |
| *daf-16(mu86)* | 14.5 | 200 | 5 | 23 |  |  |  |
| *daf-16(mu86);smo-1(RNAi)* | 14 | 249 | 12 | 20.5 | <0.0001 | compared to *daf-16(mu86)* |  |
| Experiment #13 | | | | | | | |
| wild type | 17 | 182 | 8 | 27 |  |  |  |
| *smo-1(RNAi)* | 15 | 206 | 9 | 20 | <0.0001 | compared to wild type |  |
| *daf-16(mu86)* | 15 | 177 | 8 | 20.5 |  |  |  |
| *daf-16(mu86);smo-1(RNAi)* | 14 | 213 | 10 | 17 | <0.0001 | compared to *daf-16(mu86)* | graph shown in Figure 4A |
| *daf-2(e1370)* | 34 | 111 | 3 | 56 |  |  |  |
| *daf-2(e1370);smo-1(RNAi)* | 29 | 204 | 8 | 38 | <0.0001 | compared to *daf-2(e1370)* |  |
| *daf-2(e1370);ulp-1(RNAi)* | 39 | 175 | 10 | 60 | <0.0001 | compared to *daf-2(e1370)* |  |
| Experiment #14 | | | | | | | |
| wild type | 15 | 125 | 4 | 27 |  |  |  |
| *ulp-4(RNAi)* | 17 | 136 | 9 | 30 | 0.0067 | compared to wild type |  |
| *daf-2(e1370)* | 36 | 184 | 10 | 57 |  |  |  |
| *daf-2(e1370);ulp-1(RNAi)* | 41 | 164 | 8 | 61 | <0.0001 | compared to *daf-2(e1370)* |  |
| Experiment #15 | | | | | | | |
| wild type | 17 | 190 | 5 | 24.5 |  |  |  |
| *smo-1(RNAi)* | 16 | 193 | 5 | 21 | <0.0001 | compared to wild type | graph shown in Figure 2A |
| *daf-2(e1370)* | 42 | 167 | 8 | 55.5 |  |  |  |
| *daf-2(e1370);smo-1(RNAi)* | 29 | 207 | 6 | 37.5 | <0.0001 | compared to *daf-2(e1370)* |  |
| FGP14 | 21 | 170 | 6 | 29.5 | <0.0001 | compared to wild type | graph shown in Figure 2B |
| FGP14;*daf-16(RNAi)* | 19 | 167 | 12 | 24.5 | <0.0001 | compared to FGP14 |  |
| FGP14;*skn-1(RNAi)* | 15 | 188 | 12 | 19.5 | <0.0001 | compared to FGP14 |  |
| Experiment #16 | | | | | | | |
| wild type | 15 | 149 | 6 | 25 |  |  |  |
| *smo-1(RNAi)* | 14 | 128 | 13 | 20 | <0.0001 | compared to wild type |  |
| *ulp-1(RNAi)* | 16 | 155 | 3 | 25.5 | 0.5432 | compared to wild type |  |
| *ife-2(ok306)* | 16 | 174 | 4 | 36 |  |  |  |
| *ife-2(ok306);ulp-1(RNAi)* | 18 | 162 | 2 | 33 | 0.0274 | compared to *ife-2(ok306)* |  |
| *daf-2(e1370)* | 28 | 148 | 2 | 52 |  |  |  |
| *daf-2(e1370);smo-1(RNAi)* | 21 | 168 | 6 | 37.5 | <0.0001 | compared to *daf-2(e1370)* | graph shown in Figure 4B |
| *daf-2(e1370);ulp-1(RNAi)* | 30 | 158 | 4 | 56 | 0.0006 | compared to *daf-2(e1370)* | graph shown in Figure 4B |
| Experiment #17 | | | | | | | |
| *unc-119*(+) | 14 | 145 | 14 | 31 |  |  |  |
| p*_vha-6_smo-1* | 16 | 159 | 13 | 31 | 0.0366 | compared to *unc-119*(+) |  |
| p*_rab-3_smo-1* | 16 | 100 | 18 | 29 | 0.6463 | compared to *unc-119*(+) |  |
| Experiment #18 | | | | | | | |
| *unc-119*(+) | 21 | 135 | 10 | 31 |  |  |  |
| p*_vha-6_smo-1* | 23 | 173 | 8 | 36 | 0.0065 | compared to *unc-119*(+) | graph shown in Figure 2E |
| p*_rab-3_smo-1* | 17 | 160 | 16 | 36 | 0.9164 | compared to *unc-119*(+) | graph shown in Figure 2F |
| Experiment #19 | | | | | | | |
| wild type at 25°C | 13 | 206 | 22 | 23 |  |  |  |
| *ulp-1(RNAi)* at 25°C | 15 | 235 | 17 | 22 | 0.0212 | compared to wild type | graph shown in Figure S1D |
| Experiment #20 | | | | | | | |
| wild type at 25°C | 13 | 182 | 12 | 23 |  |  |  |
| *ulp-1(RNAi)* at 25°C | 13 | 190 | 11 | 21 | 0.4819 | compared to wild type |  |
| Experiment #21 | | | | | | | |
| wild type at 25°C | 13 | 209 | 11 | 20 |  |  |  |
| *ulp-1(RNAi)* at 25°C | 14 | 228 | 15 | 22 | 0.2966 | compared to wild type |  |
| Experiment #22 | | | | | | | |
| wild type at 25°C | 10 | 188 | 13 | 22 |  |  |  |
| *ulp-1(RNAi)* at 25°C | 14 | 194 | 13 | 22 | <0.0001 | compared to wild type |  |
| Experiment #23 | | | | | | | |
| wild type on paraquat (PQ) | 4 | 96 | 5 | 6.5 |  |  |  |
| *smo-1(RNAi)* on PQ | 4 | 111 | 5 | 6 | 0.0092 | compared to wild type |  |
| FGP14 on PQ | 7 | 88 | 6 | 12.5 | <0.0001 | compared to wild type |  |
| Experiment #24 | | | | | | | |
| wild type on paraquat (PQ) | 5 | 107 | 8 | 9.5 |  |  |  |
| *smo-1(RNAi)* on PQ | 4 | 112 | 4 | 8 | 0.0108 | compared to wild type |  |
| FGP14 on PQ | 7 | 80 | 9 | 15 | <0.0001 | compared to wild type |  |
| Experiment #25 | | | | | | | |
| wild type on paraquat (PQ) | 4 | 91 | 9 | 7 |  |  |  |
| *smo-1(RNAi)* on PQ | 4 | 76 | 8 | 6.5 | 0.21 | compared to wild type | graph shown in Figure 3C |
| *skn-1(RNAi)* on PQ | 3 | 128 | 5 | 5 | <0.0001 | compared to wild type | graph shown in Figure 3C |
| FGP14 on PQ | 5 | 67 | 12 | 11 | 0.0004 | compared to wild type | graph shown in Figure 3C |
| FGP14;*skn-1(RNAi)* on PQ | 4 | 71 | 8 | 6.5 | 0.0001 | compared to FGP14 | graph shown in Figure 3C |
| Experiment #26 | | | | | | | |
| wild type on paraquat (PQ) | 4 | 86 | 5 | 6.5 |  |  |  |
| *smo-1(RNAi)* on PQ | 4 | 103 | 6 | 7 | 0.8862 | compared to wild type |  |
| *skn-1(RNAi)* on PQ | 3 | 110 | 8 | 5.5 | <0.0001 | compared to wild type |  |
| FGP14 on PQ | 6 | 58 | 10 | 12.5 | <0.0001 | compared to wild type |  |
| FGP14;*skn-1(RNAi)* on PQ | 3 | 64 | 10 | 6 | <0.0001 | compared to FGP14 |  |
| Experiment #27 | | | | | | | |
| wild type after heat shock (HS) | 10 | 105 | 11 | 17 |  |  |  |
| *smo-1(RNAi)* after HS | 8 | 114 | 12 | 13.5 | 0.0508 | compared to wild type | graph shown in Figure 3D |
| FGP14 after HS | 11 | 87 | 15 | 22 | <0.0001 | compared to wild type | graph shown in Figure 3D |
| Experiment #28 | | | | | | | |
| wild type after heat shock (HS) | 9 | 96 | 12 | 13.5 |  |  |  |
| *smo-1(RNAi)* after HS | 9 | 104 | 10 | 13.5 | 0.7065 | compared to wild type |  |
| FGP14 after HS | 12 | 49 | 16 | 19.5 | <0.0001 | compared to wild type |  |
| Experiment #29 | | | | | | | |
| wild type | 17 | 76 | 13 | 25 |  |  |  |
| wild type on NAC | 14 | 78 | 22 | 25 | 0.0521 | compared to wild type | graph shown in Figure S4F |
| FGP14 | 21 | 76 | 15 | 29 |  |  |  |
| FGP14 on NAC | 17 | 80 | 20 | 25.5 | <0.0001 | compared to FGP14 | graph shown in Figure S4F |
